# Supplementary material for: SMAD4 mutations causing Myhre syndrome are under positive selection in the male germline
Source: Am J Hum Genet. 2024 Aug 7;111(9):1953–69. doi: 10.1016/j.ajhg.2024.07.006 (PMC11444041; doi:10.1016/j.ajhg.2024.07.006)
Supplement: Document S1. Figures S1–S7 [file mmc1.pdf]

The American Journal of Human Genetics, Volume *111*

Supplemental information

SMAD4 mutations causing Myhre syndrome are under positive selection in the male germline

Katherine A. Wood, R Spencer Tong, Marialetizia Motta, Viviana Cordeddu, Eleanor R. Scimone, Stephen J. Bush, Dale W. Maxwell, Eleni Giannoulatou, Viviana Caputo, Alice Traversa, Cecilia Mancini, Giovanni B. Ferrero, Francesco Benedicenti, Paola Grammatico, Daniela Melis, Katharina Steindl, Nicola Brunetti-Pierri, Eva Trevisson, Andrew OM. Wilkie, Angela E. Lin, Valerie Cormier-Daire, Stephen RF. Twigg, Marco Tartaglia, and Anne Goriely

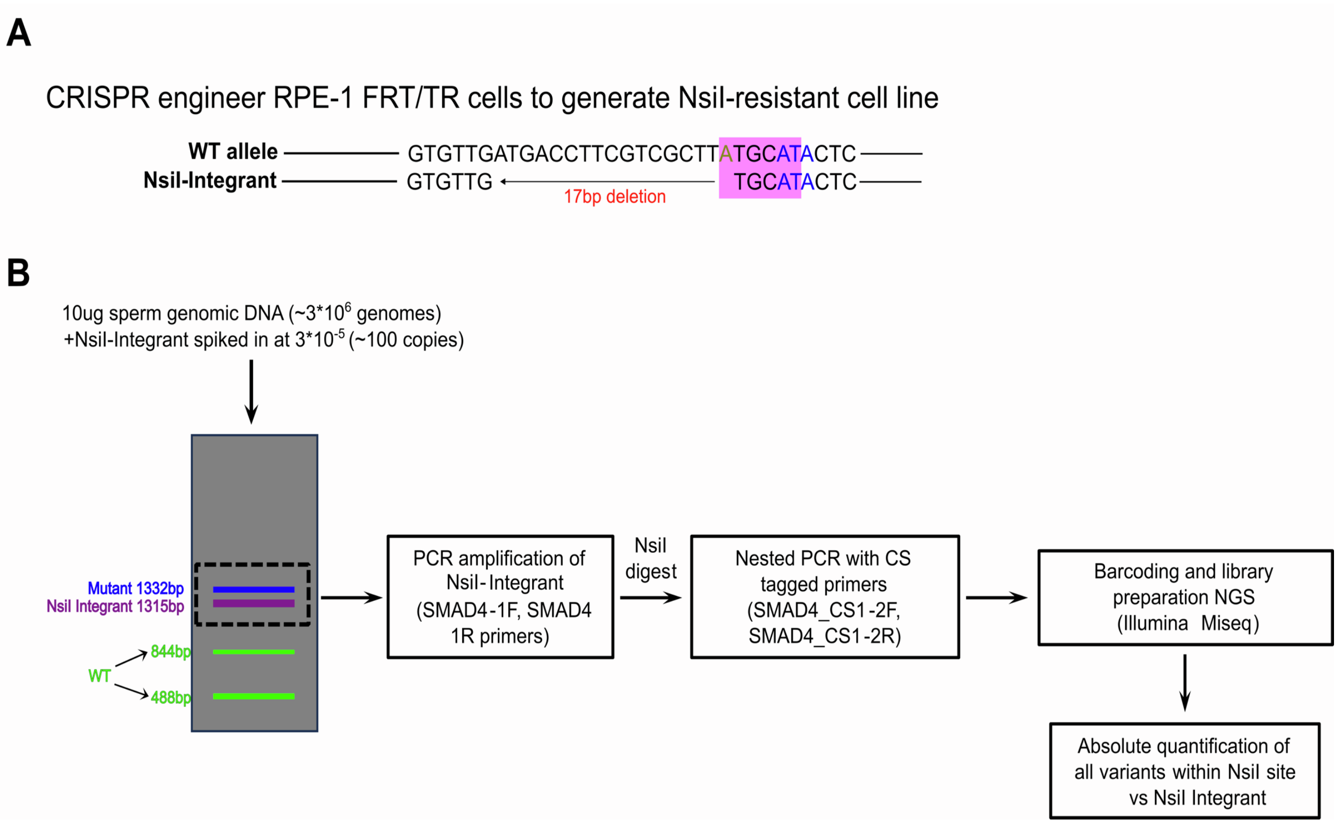

Figure S1. Overview of the NsiI RED-PCR assay to enrich mutant sequences at position c.1494_1499 of *SMAD4.* (**A**) Sequence context of the NsiI-integrant following CRISPR-Cas engineering, a heterozygous clone with a 17-bp deletion (c.1478_1494del). The NsiI site is boxed in purple and the Ile500 codon is highlighted in blue. The engineered mutant allele disrupts the NsiI restriction site (the final base of the deletion encompasses the first base of the NsiI cut site (position c.1494A) making the CRISPR allele resistant to enzyme digestion. (**B**) Overview of the RED-PCR design involving a double NsiI and PstI digestion of 10 µg of human gDNA (blood or sperm), supplemented with ~100 copies of mutant allele of the NsiI-Integrant clone, separation of the NsiI-resistant fragments by gel electrophoresis, followed by two rounds of PCR amplification, barcoding of individual samples and ultra-deep Illumina sequencing.

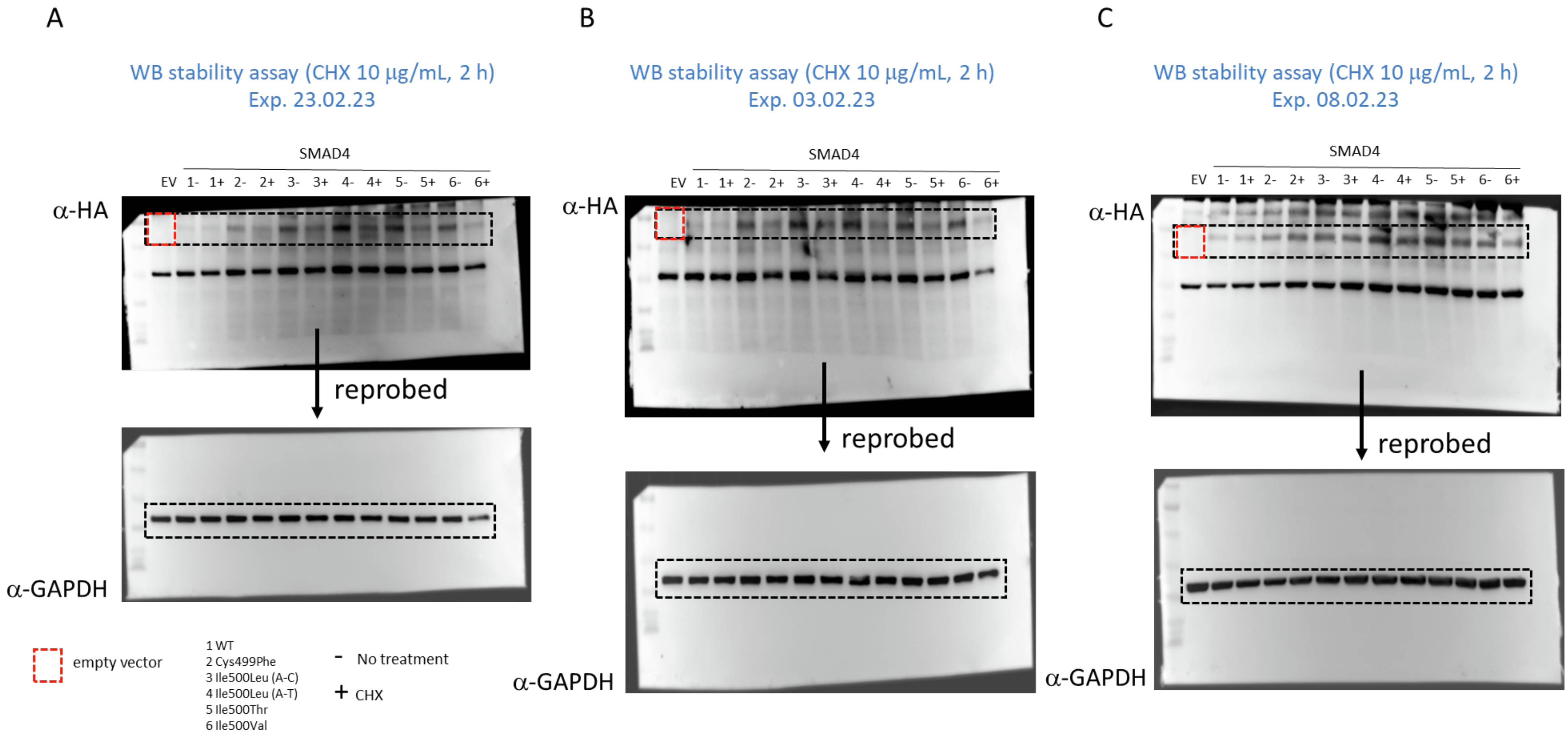

Figure S2. Uncropped pictures of the western blotting membranes used for the densitometric analysis of SMAD4 protein levels (SMAD4 stability assay) presented in **Figure 3B**. (**A**), (**B**) and (**C**) refer to the three independent experiments performed. For each membrane, the extent of the cropped region is shown. For experimental conditions and antibody specifications please refer to the Materials and Methods section.

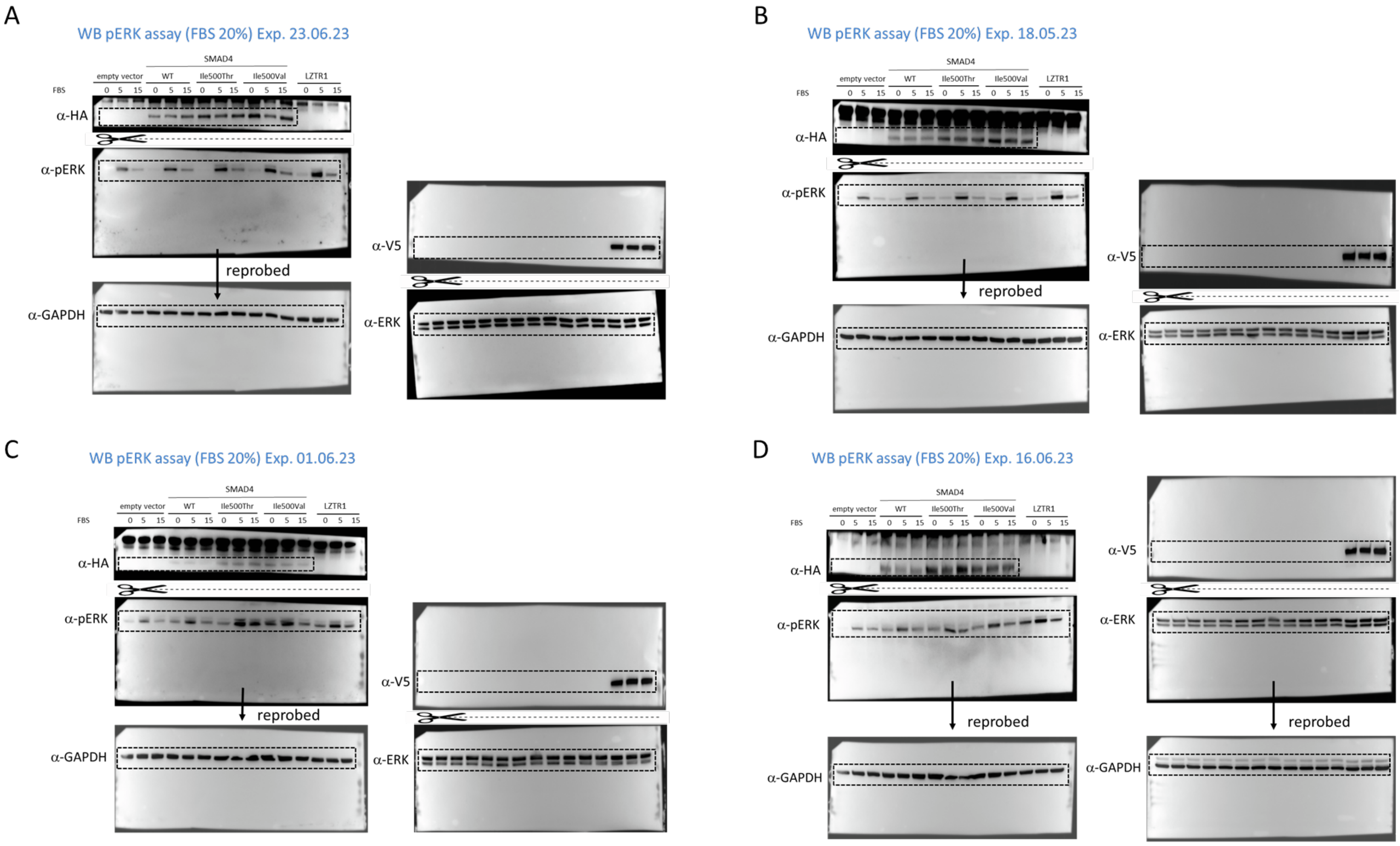

Figure S3. Uncropped pictures of western blotting membranes used for densitometric analysis of ERK1/2 phosphorylation levels at Thr202/Thr185 and Tyr204/Tyr187 (pERK assay) presented in **Figure 3C**. (**A**), (**B**), (**C**) and (**D**) refer to the four independent experiments performed. For each membrane, the extent of the cropped region is shown. Membranes were cut to enable blotting for multiple antibodies. For experimental conditions and antibodies specifications please refer to the Materials and Methods section.

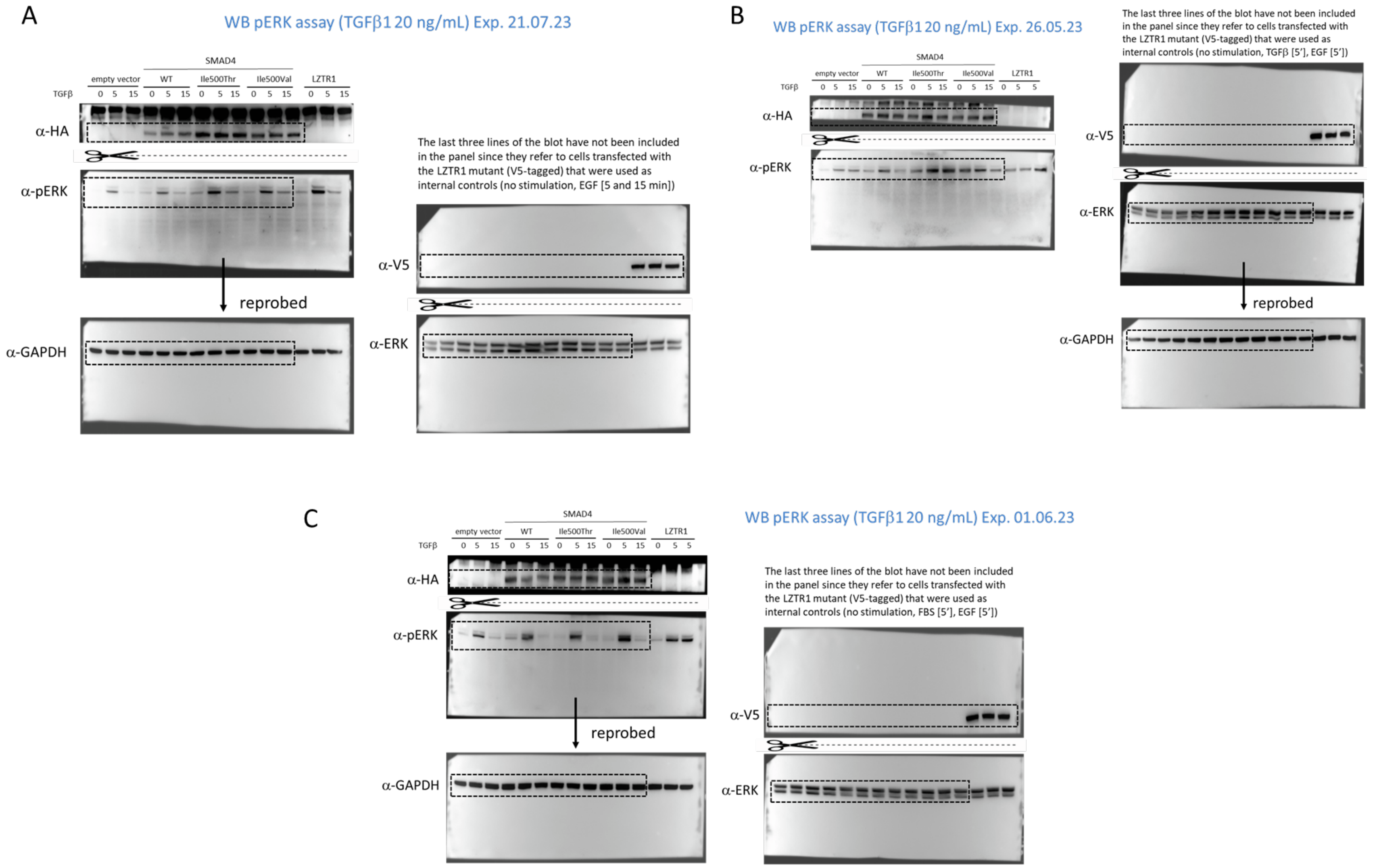

Figure S4. Uncropped pictures of western blotting membranes used for densitometric analysis of ERK1/2 phosphorylation levels at Thr202/Thr185 and Tyr204/Tyr187 (pERK assay) (TGFβ1 stimulation) presented in **Figure 3D**. (**A**), (**B**) and (**C**) refer to the three independent experiments performed. For each membrane, the extent of the cropped region is shown. Membranes were cut to enable blotting for multiple antibodies. For experimental conditions and antibodies specifications please refer to the Materials and Methods section.

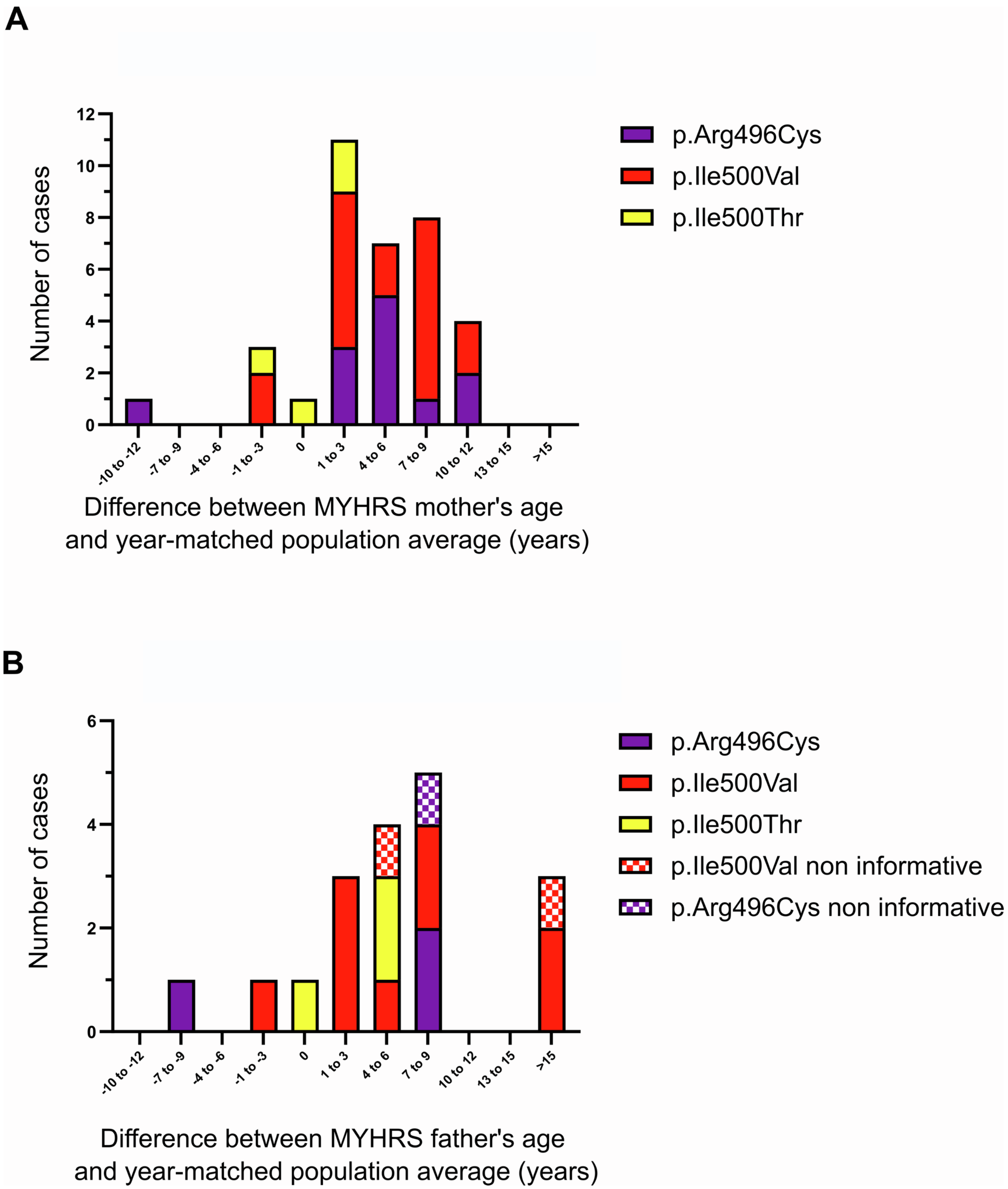

Figure S5. Parental age effects in MYHRS cohorts. (**A**) Maternal age excess observed for probands with confirmed MYHRS *de novo* variants (as indicated on the figure) for a USA cohort (n = 35). (**B**) Excess paternal age observed in MYHRS families for a European (EU) cohort with confirmed parental origin of the *de novo* mutation. The X-axis represents the difference of paternal ages between MYHRS fathers and the year-match fatherhood average in England and Wales (UK). Note we had access to age data for 17 EU MYHRS families and among them 14 had proven paternal origin excess age (plain colors). The 3 uninformative families are indicated in dotted colors (see **Table S2**).

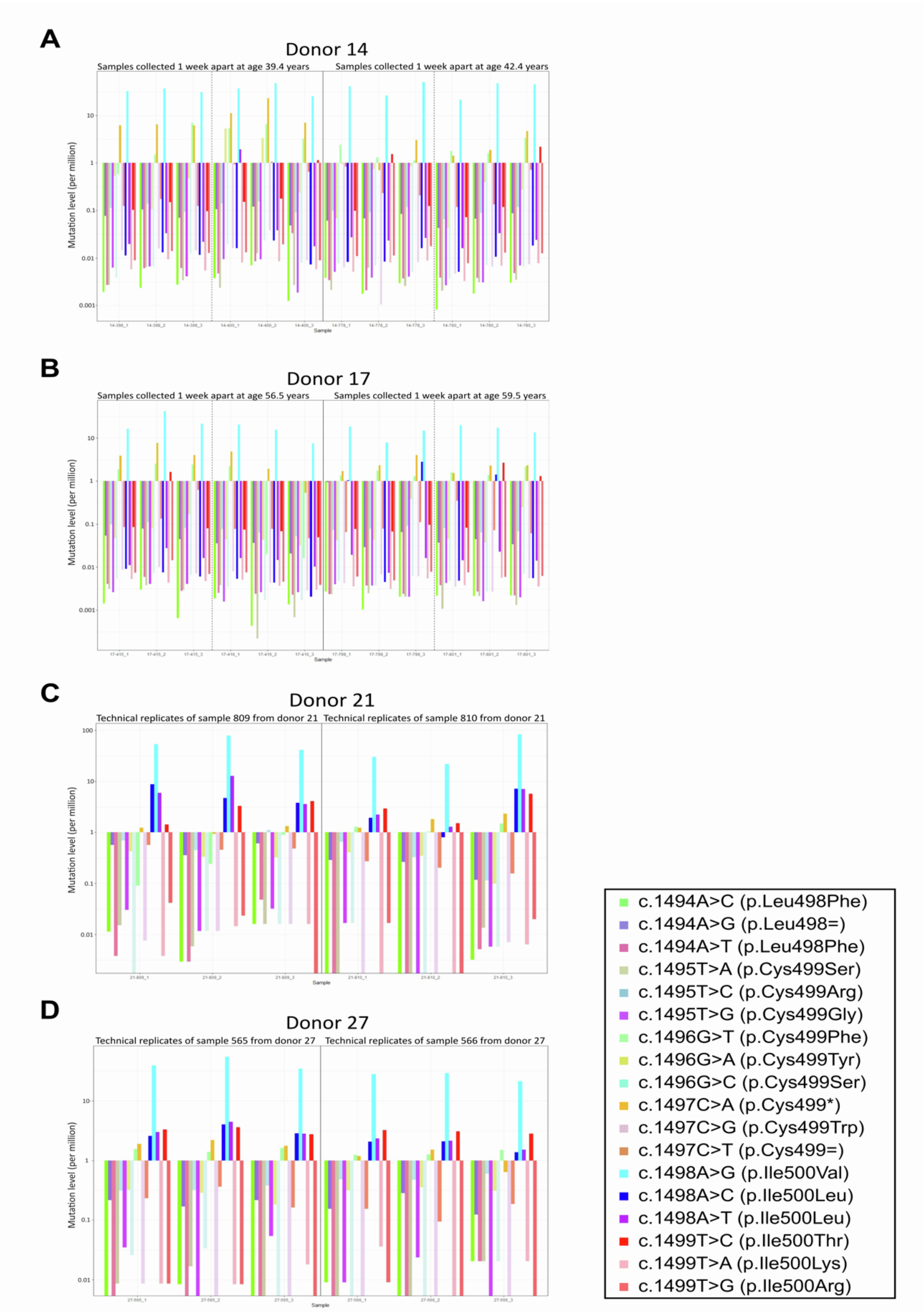

Figure S6. Reproducibility of the NsiI RED_PCR assay. The mutation levels of each substitution at *SMAD4* c.1494_1499 were quantified by the NsiI RED_PCR assay for independent biological semen samples from 4 different donors. For each sample, 3 technical replicates (underscores 1-3 on the X-axis) were processed in parallel. (**A-B**) For donors 14 and 17, four sets of samples are shown, two samples were taken a week apart and another set of two samples from the same donor three years later. (**C-D**) For donors 21 and 27, two sets of samples were collected within a week of each other (figure relates to data presented in **Table S4**).

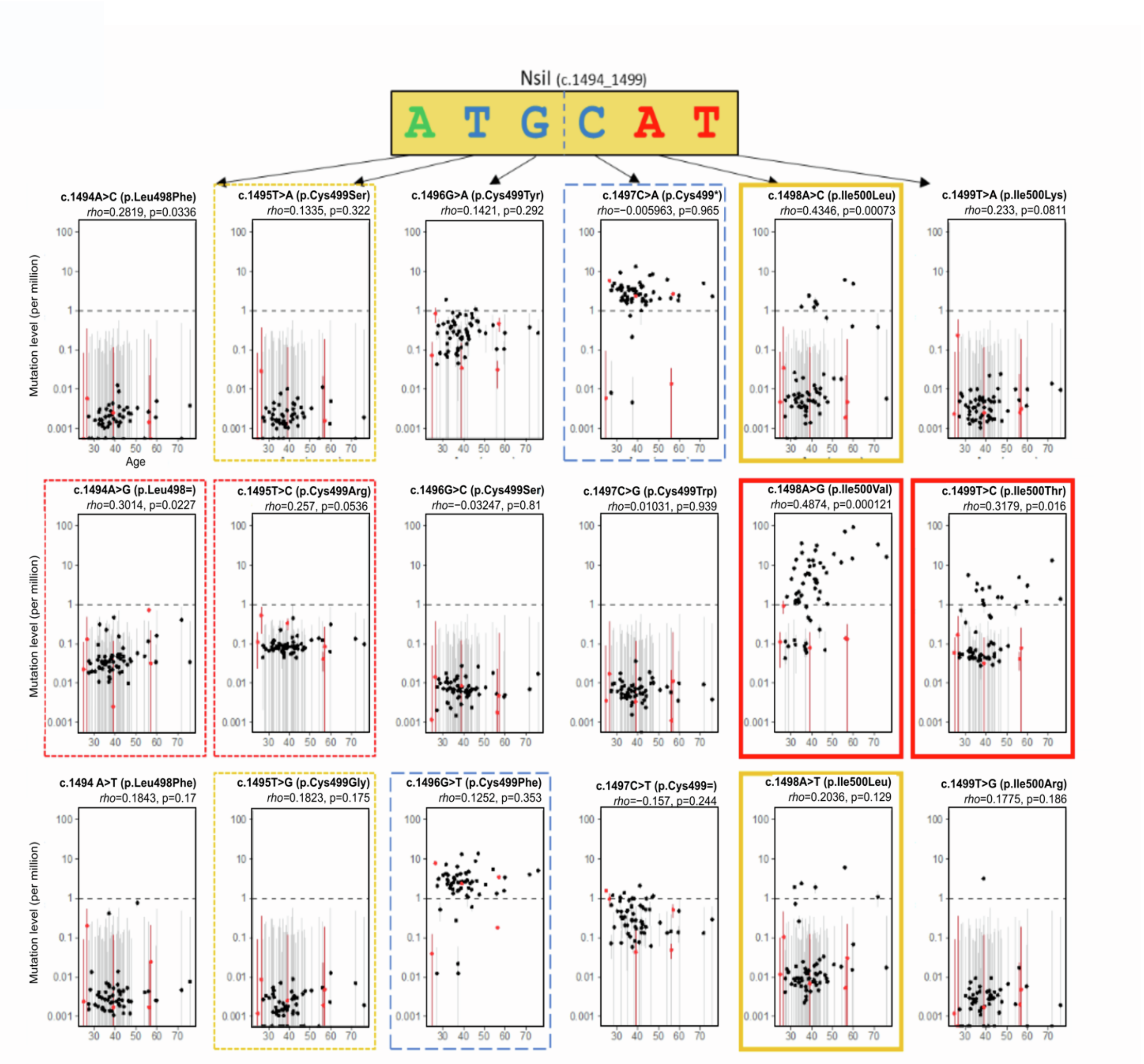

Figure S7. Quantification of mutation levels within the NsiI site in sperm and blood samples. Each of the 18 substitutions at *SMAD4* c.1494_1499 from 57 sperm (black) and 5 blood (red) samples (from donors aged 24.8 - 75.9 years, age shown on X-axis) are shown. Panels have been organized according to the substitutions within the NsiI site (orange box at the top, note the palindromic nature of the site, indicated by the dotted blue line within the box). Estimated mutation levels following the RED_PCR enrichment strategy are plotted as the mean of 3 independent technical replicates and their 95% binomial CI on a log10 scale (Y-axis). The dotted horizontal black lines represent the theoretical 10-6 limit of detection of the RED_PCR assay. The mutations found to be enriched in sperm have been boxed (red box for the two known MYHRS variants c.1488A>G and c.1499T>C) and orange for the two substitutions encoding p.Ile500Leu). Moreover, their cognate/reciprocal positions within the palindromic site are highlighted by dotted boxes of matching colors. Also note the p.Cys499Phe/Stop substitutions (dotted blue boxes) are cognate (c.1496G>T | c.1497C>A) substitutions and show a symmetrical pattern. A rho value representing the correlation between mutation level and donor age is shown above each panel. Individual data are provided in **Table S5** and summarized in **Table S6**.
