# Supplementary material for: SMAD4 mutations causing Myhre syndrome are under positive selection in the male germline
Source: Am J Hum Genet. 2024 Aug 7;111(9):1953–69. doi: 10.1016/j.ajhg.2024.07.006 (PMC11444041; doi:10.1016/j.ajhg.2024.07.006)
Supplement: Document S2. Article plus supplemental information [file mmc3.pdf]

SMAD4 mutations causing Myhre syndrome are under positive selection in the male germline

Authors

Katherine A. Wood, R Spencer Tong, Marialetizia Motta, ..., Stephen RF. Twigg, Marco Tartaglia, Anne Goriely

Correspondence

marco.tartaglia@opbg.net (M.T.), anne.goriely@imm.ox.ac.uk (A.G.)

Activating RAS-MAPK mutations occurring spontaneously in human spermatogonia cause clonal expansion of these stem cells with age, explaining the high birth prevalence of the associated disorders. We show that Myhre syndrome-causing mutations in *SMAD4*, a gene operating outside the canonical RAS-MAPK signaling pathway, are positively selected in the male germline.

Wood et al., 2024, The American Journal of Human Genetics *111*, 1953–1969

September 5, 2024 © 2024 The Authors.

https://doi.org/10.1016/j.ajhg.2024.07.006

CellPress

SMAD4 mutations causing Myhre syndrome are under positive selection in the male germline

Katherine A. Wood,[1,2,3,22] R Spencer Tong,[1,2,3,22] Marialetizia Motta,[4,22] Viviana Cordeddu,[5] Eleanor R. Scimone,[6] Stephen J. Bush,[1,2,3,20] Dale W. Maxwell,[1,2,3] Eleni Giannoulatou,[7,8] Viviana Caputo,[9] Alice Traversa,[9] Cecilia Mancini,[4] Giovanni B. Ferrero,[10] Francesco Benedicenti,[11] Paola Grammatico,[12] Daniela Melis,[13] Katharina Steindl,[14] Nicola Brunetti-Pierri,[15,16,17] Eva Trevisson,[18] Andrew OM. Wilkie,[1,2,3] Angela E. Lin,[6] Valerie Cormier-Daire,[19] Stephen RF. Twigg,[1,2,3] Marco Tartaglia,[4,21,*] and Anne Goriely[1,2,3,21,*]

Summary

While it is widely thought that *de novo* mutations (DNMs) occur randomly, we previously showed that some DNMs are enriched because they are positively selected in the testes of aging men. These “selfish” mutations cause disorders with a shared presentation of features, including exclusive paternal origin, significant increase of the father’s age, and high apparent germline mutation rate. To date, all known selfish mutations cluster within the components of the RTK-RAS-MAPK signaling pathway, a critical modulator of testicular homeostasis. Here, we demonstrate the selfish nature of the *SMAD4* DNMs causing Myhre syndrome (MYHRS). By analyzing 16 informative trios, we show that MYHRS-causing DNMs originated on the paternally derived allele in all cases. We document a statistically significant epidemiological paternal age effect of 6.3 years excess for fathers of MYHRS probands. We developed an ultra-sensitive assay to quantify spontaneous MYHRS-causing *SMAD4* variants in sperm and show that pathogenic variants at codon 500 are found at elevated level in sperm of most men and exhibit a strong positive correlation with donor’s age, indicative of a high apparent germline mutation rate. Finally, we performed *in vitro* assays to validate the peculiar functional behavior of the clonally selected DNMs and explored the basis of the pathophysiology of the different *SMAD4* sperm-enriched variants. Taken together, these data provide compelling evidence that *SMAD4*, a gene operating outside the canonical RAS-MAPK signaling pathway, is associated with selfish spermatogonial selection and raises the possibility that other genes/pathways are under positive selection in the aging human testis.

Introduction

Understanding the factors that impact the prevalence of *de novo* mutations (DNMs) in the human genome is critical for our understanding of genetic disorders, genomic diversity, and evolution. In humans, spontaneous germline point mutations are estimated to occur at a rate of 1.2×10^{-8} per nucleotide.[1,2] These mutations tend to be paternal in origin, with approximately 75% of DNMs occurring on the paternally derived chromosomes.[1,3,4] This gender bias in DNM load likely originates from the continued mitotic replications of spermatogonial stem cells (SSCs) throughout male reproductive life. For most DNMs, the age of the father at conception has a modest (and linear) impact on mutation rate, accounting for approximately two additional DNMs in a child per additional year of age of the father at conception.[1,5,6] However, for some congenital disorders, which are caused by “selfish” or paternal age effect (PAE) DNMs, a different presentation pattern of mutation is evident.[2,7] PAE disorders share three unusual characteristics: (1) the causative mutations are exclusively of paternal origin, indicating that the original mutational event occurs in the male germline during spermatogenesis; (2) the disorders are associated with a significant epidemiological paternal age effect, where fathers are, on average, two to seven

[1]MRC Weatherall Institute of Molecular Medicine, Oxford OX39DS, UK; [2]Nuffield Division of Clinical Laboratory Sciences, Radcliffe Department of Medicine, University of Oxford, Oxford OX39DS, UK; [3]NIHR Oxford Biomedical Research Centre, Oxford OX39DU, UK; [4]Molecular Genetics and Functional Genomics, Bambino Gesù Children’s Hospital, IRCCS, 00146 Rome, Italy; [5]Department of Oncology and Molecular Medicine, Istituto Superiore di Sanità, 00161 Rome, Italy; [6]Medical Genetics, Mass General Brigham, Harvard Medical School, Harvard University, Boston, MA 02114, USA; [7]Victor Chang Cardiac Research Institute, Darlinghurst, Sydney, NSW 2010, Australia; [8]School of Clinical Medicine, St Vincent’s Healthcare Clinical Campus, Faculty of Medicine and Health, UNSW Sydney, Sydney, NSW 2052, Australia; [9]Department of Experimental Medicine, Sapienza University, 00161 Rome, Italy; [10]Department of Clinical and Biological Science, University of Torino, 10126 Turin, Italy; [11]Genetic Counseling Service, Regional Hospital of Bolzano, 39100 Bolzano, Italy; [12]Department of Experimental Medicine, San Camillo-Forlanini Hospital, Sapienza University, 00152 Rome, Italy; [13]Department of Medicine, Surgery and Dentistry, University of Salerno, 84081 Salerno, Italy; [14]Institute of Medical Genetics, University of Zurich, 8952 Schlieren-Zurich, Switzerland; [15]Department of Translational Medicine, Federico II University, 80131 Naples, Italy; [16]Telethon Institute of Genetics and Medicine, Pozzuoli, Italy; [17]Scuola Superiore Meridionale (SSM, School of Advanced Studies), Genomics and Experimental Medicine Program, University of Naples Federico II, Naples, Italy; [18]Department of Women’s and Children’s Health, University of Padova, 35128 Padua, Italy; [19]Université Paris Cité, Service de Médecine Génomique des Maladies Rares, INSERM UMR 1163, Institut Imagine, Hôpital Necker-Enfants Malades, 75015 Paris, France
[20]Present address: School of Automation Science and Engineering, Xi’an Jiaotong University, Xi’an 710049, China
[21]Senior authors
[22]These authors contributed equally
*Correspondence: marco.tartaglia@opbg.net (M.T.), anne.goriely@imm.ox.ac.uk (A.G.)
https://doi.org/10.1016/j.ajhg.2024.07.006.
© 2024 The Authors. This is an open access article under the CC BY license (http://creativecommons.org/licenses/by/4.0/).

years older than the population mean; and (3) a high apparent germline mutation rate of the causative mutations is observed (up to 1,000 times higher than background), translating into an elevated birth prevalence and narrow mutational spectrum for specific recurrent mutations.[7]

PAE mutations are well documented in *FGFR2* (MIM: 176943), *FGFR3* (MIM: 134934), *HRAS* (MIM: 190020), *PTPN11* (MIM: 176876), and *RET* (MIM: 164761); such mutations cause dominant disorders and encode gain-of-function (GoF) missense substitutions.[7] Previously, we have proposed that SSCs acquire rare spontaneous PAE mutations during mitotic replication in the testes. These GoF mutations are then positively selected, resulting in progressive clonal expansion of mutant spermatogonia as men age, explaining the high apparent birth rate observed for classical PAE disorders, such as achondroplasia (MIM: 100800), Apert (MIM: 101200), and Noonan (MIM: 163950) syndromes. So far, 13 genes have been directly implicated in selfish selection and are all functionally related to the RTK-RAS-MAPK signaling cascade,[8–17] a central pathway controlling SSC homeostasis.[18,19] The selfish nature of PAE mutations has mainly been studied by looking for local enrichment in human testes and/or quantifying specific mutation levels in sperm from men of different ages.[4]

Other genes may be under positive selection in human testes. To identify new selfish candidate genes, we reasoned that given that these mutations are selected in aging testes and are present at elevated levels in the semen of most men, they would be expected to recur more frequently as DNMs in patient cohorts. The analysis of whole-exome sequencing (WES) data from more than 31,000 parent-child trios with a child affected by a developmental disorder had previously identified the most commonly recurring DNMs.[20] Among the top 15 were two known pathogenic mutations—c.1486C>T (p.Arg496Cys) and c.1498A>G (p.Ile500Val) in *SMAD4* ([MIM: 600993] GenBank: NM_005359.6)—which cause Myhre syndrome (MYHRS [MIM: 139210]).[21–23] Independently, we identified both variants, as well as the c.1500A>G (another missense change implicated in MYHRS, encoding p.Ile500Met) in the same gene in screens of testicular biopsies,[24] suggesting that MYHRS-causing *SMAD4* mutations may be under positive selection in human testes.

MYHRS is a rare autosomal-dominant congenital disorder characterized by intellectual disability, dysmorphic facial features, skeletal anomalies, and cardiovascular defects,[25] which is caused by heterozygous mutations within either one of two codons (Arg496 and Ile500) of *SMAD4*.[26] SMAD4 is an essential effector of the TGF-β/BMP/activin pathway, acting as a key mediator of signal transduction.[27] It is characterized by an *N*-terminal MH1 domain, responsible for binding to DNA, a SMAD4 activation domain (SAD) necessary for its transcriptional activity, and a C-terminal MH2 domain, mediating SMAD4 transcriptional activity and interaction with other SMAD proteins.[28] All resolved MYHRS cases to date have one of four heterozygous missense variants in the two affected codons (c.1486C>T [p.Arg496Cys], c.1498A>G [p.Ile500Val], c.1499T>C [p.Ile500Thr], or c.1500A>G [p.Ile500Met]) within the MH2 domain of SMAD4. These substitutions have been predicted to act via a GoF mechanism, possibly through altered stability of the SMAD heterotrimer and/or reduced ubiquitination of SMAD4,[21–23,29] although a dominant-negative model has also been proposed.[30] In contrast, nonsense and truncating loss-of-function (LoF) germline variants in *SMAD4* are associated with juvenile polyposis syndrome (JPS [MIM: 174900]) and JPS-hereditary hemorrhagic telangiectasia (JPHT [MIM: 175050]).[31,32] The LoF variants are widely distributed throughout the length of the protein although they are distinctly absent around codons Arg496 and Ile500.[33] Similarly, gene deletions of *SMAD4* and somatic inactivating intragenic lesions found throughout the gene length frequently occur in various cancers including pancreatic duct carcinomas (COSMIC).[34] Single-cell (sc)RNA-seq analysis has shown that *SMAD4* is expressed in SSCs and that TGF-β/BMP/activin signaling is critical for SSC homeostasis.[35–37] Consistent with this, *SMAD4* is expressed in SSCs (Human Protein Atlas, www.proteinatlas.org).[35,38] Taken together with the narrow mutational spectrum and likely pathogenic mechanism associated with MYHRS, our preliminary findings identify *SMAD4* as a candidate for selfish selection in human testes.

Here, we tested the hypothesis that MYHRS-associated *SMAD4* variants are positively selected in the male germline by asking whether these mutations fulfill the three distinctive features of PAE disorders. To assess this, first, we performed parent-of-origin studies on MYHRS-affected family trios and showed that for all informative cases, the *SMAD4* DNMs are present on the paternally derived allele. Secondly, we analyzed parental age data from a well-characterized cohort of individuals with MYHRS to quantify the PAE associated with *SMAD4* DNMs. Thirdly, we developed an ultra-sensitive assay to directly quantify spontaneous *SMAD4* mutation levels in the semen of men of different ages, and we show that the missense changes resulting in p.Ile500Val and p.Ile500Thr are present at elevated levels in the sperm of many men and increase in prevalence with the age of the donor. We also show that the p.Ile500Leu change (caused by either c.1498A>T or c.1498A>C substitutions), not previously reported in association with MYHRS, is under positive selection in the male germline. Finally, we validated the differential putative functional impact of the *SMAD4* selfish variants in terms of transcriptional activity, protein stability, and intracellular signaling. Overall, our data show that MYHRS-associated *SMAD4* variants fulfill the presentation criteria of classical PAE disorders, and so represent selfishly selected mutations affecting a signaling pathway outside the canonical RAS-MAPK pathway.

Subjects, material, and methods

Biological samples

DNA from blood samples for 18 individuals with molecularly confirmed clinical diagnosis of MYHRS and their unaffected parents were collected in accordance with ethical standards of institutional revision committees and with informed consent, following approval by the local Institutional Ethical Committee of the Ospedale Pediatrico Bambino Gesù (1702_OPBG_2018), Rome, Italy and Azienda Ospedaliera Universitaria Federico II, Naples, Italy (48/16).

For the restriction enzyme digestion-PCR (RED-PCR) ultrasensitive detection assay, sperm and blood samples were collected from anonymous donors (aged 24–75 years). Written informed consent was obtained from all donors and collected with the permission of the North East - Newcastle & North Tyneside 1 Research Ethics Committee (22/NE/0025).

DNA was extracted from sperm and blood and quantified using standard fluorometric methods as previously described.[8]

Haplotype-phasing of *de novo* MYHRS-causing *SMAD4* mutations

Establishing the parental origin of MYHRS DNMs required the presence of an informative single-nucleotide polymorphism (SNP) near the *SMAD4* pathogenic variant in the proband. To determine whether the proband carried a heterozygous SNP that allows us to distinguish the two parental alleles, up to 11 overlapping fragments covering a region of ~4.4 kb around the mutation sites of *SMAD4* (exon 11) were PCR amplified with GoTaq DNA Polymerase (Promega) (primers reported in Table S1), purified (NucleoSpin Gel and PCR Clean-up, Macherey-Nagel), and sequenced (ABI BigDye Terminator Cycle Sequencing kit v.3.1, Applied Biosystems) on an ABI Prism 3500 Genetic Analyzer, as per manufacturer's protocol. Following identification of heterozygous SNPs in probands, parental DNA was sequenced to establish informativeness. For those families with an informative SNP, a genomic fragment encompassing the *SMAD4* pathogenic DNM and the SNP was amplified from the proband DNA (Table S1) and cloned using TA Cloning Kit (Invitrogen), as per manufacturer's protocol. To distinguish which haplotype (maternal or paternal) carried the causative variant, dideoxy-sequencing analysis of the cloned fragments was performed. A minimum of 10 clones were sequenced for each proband.

For 4 families (ID 18, 19, 21, and 22), long-read sequencing using Oxford Nanopore Technology covering two genomic fragments (region 1, approximately 10 kb; region 2, approximately 7 kb) (Table S2) was used to determine the phase, following a previously described approach,[39] which allowed identification of informative SNP(s) in each individual family trio and haplotyping of the DNM to take place in a single sequencing reaction.

Epidemiological parental age effects

We collected age data for 35 family trios for which the affected proband was born in the USA, had a confirmed *de novo SMAD4* mutation, and had a clinical diagnosis of MYHRS. This research was approved by the MGH (Mass General Brigham, Harvard Medical School) Institutional Review Board under protocols #2015P001173 and #2000P001531. We compared the difference in age (at the time of birth) of the parents with the year-matched USA population average. To obtain the mean age of parenthood in the USA, we used de-identified data of live births within mainland USA, which were obtained via the National Vital Statistics System (NVSS) at https://www.nber.org/research/data/vital-statistics-natality-birth-data (accessed 4th May 2023). The NVSS is an inter-governmental data sharing program in which participating institutions file birth certificates containing, among other information, parental demographics. The annual natality data files (spanning the years 1972–2021) were compiled into a single dataset, as previously reported.[40] For each year, we calculated the mean and standard deviation of paternal and maternal ages based on a total of 145,978,354 and 180,536,851 available birth certificates, respectively.

We also estimated the paternal age effect for the MYHRS-affected families for whom we demonstrated the paternal origin of the DNMs. For those families with paternal age data ($n = 14$), the father's age at the time of birth was compared with that of the mean paternal age from the England and Wales population data, matched for the year of birth of the proband (1997–2015). The significance of the paternal age effects was tested using a one-tailed t test.

Quantification of *SMAD4* mutation levels in sperm and blood

Mutations levels in blood and sperm DNA within the NsiI restriction site located at c.1494_1499 of *SMAD4* were analyzed using a Restriction Enzyme Digest (RED)-PCR enrichment strategy, which is illustrated on Figure S1 and is similar to that previously used in other studies.[8,9,12]

Generation of an NsiI-resistant human cell line using CRISPR-Cas9 (NsiI-Integrant)

To quantify the absolute mutation levels of spontaneous MYHRS-associated *SMAD4* variants within the *SMAD4* c.1494_1499 NsiI site, we generated an NsiI-resistant clone with a unique molecular signature by CRISPR-Cas9 engineering the human retinal pigment epithelial RPE-1-FRT/TR cell line. Briefly, the PAM site located at genomic location chr18:51,078,290–51,078,292 (GRCh38/hg38) (c.1482_1484) was targeted by designing a 20 bp guide RNA (sgRNA) sequence (SMAD4 sgRNA BbsI, Table S1) and cloned into the pSpCas9(BB)-2A-GFP (PX458) plasmid as previously described.[39,41] The construct integrity was validated by dideoxy-sequencing before electroporation (400 ng) into 2.5×10^5 RPE-1 FRT/TR cells using P3 Primary Cell Full Electroporation Buffer (Lonza) and the EA-104 program on the 4D-Nucleofector System (Lonza), in a final volume of 20 μL. Cells were plated in 1 mL

pre-warmed Dulbecco's modified Eagle's medium (DMEM) containing 10% fetal bovine serum (FBS; Sigma-Aldrich), 1% of L-glutamine, 1% Pen-Strep, 1% sodium pyruvate, and 1% non-essential amino acids (NEAA) (all reagents from Thermo Fisher Scientific, unless specified otherwise) and were allowed to recover for 48 h at 37°C with 5% CO_2. Cells were then trypsinized with 0.25% Trypsin-EDTA (1×) and selected for GFP+ in 96-well plates by fluorescence-activated cell sorting on a Sony MA900 Multi-Application Cell Sorter. Single cells were cultured for an additional 2–4 weeks under standard conditions (DMEM media mix). Clones were screened for NsiI resistance, following genomic DNA (gDNA) extraction using the QuickExtract kit (Lucigen), PCR amplification with primers SMAD4-3F and SMAD4-3R (Table S1) using Q5 polymerase for 30 cycles of 98°C (10 s), 60°C (30 s), and 72°C (30 s), and digestion with 1 U of NsiI (New England BioLabs). NsiI-resistant clones were expanded in T75 flasks for 1 week before DNA was extracted using the DNeasy Blood and Tissue kit (Qiagen) and quantified using Qubit dsDNA HS assay kit (Thermo Fisher Scientific). A mutated clone (clone 21), containing a unique 17-bp heterozygous deletion (c.1478_1494del), was verified by dideoxy sequencing and used in the RED-PCR assay (referred to as NsiI-Integrant) (Figure S1A).

Titration-reconstitution RED-PCR assay (dilution series)

To assess the sensitivity and accuracy of the NsiI RED-PCR enrichment assay, DNA samples from two MYHRS-affected individuals, heterozygous for the c.1498A>G (p.Ile500Val) or c.1499T>C (p.Ile500Thr) variant, were serially diluted (from 10^{-4} to 10^{-6}) in a titration-reconstitution RED-PCR assay. The gDNA carrying MYHRS variants are resistant to cleavage by NsiI at the position c.1494_1499ATGC<u>AT</u> (cDNA GenBank: NM_005359.6, position of A or T mutated in MYHRS underlined [see Figure 2]). 10 μg (~3 million haploid genomes) of DNA extracted from control whole blood from an anonymous donor was spiked with 0.6 ng gDNA from the NsiI-Integrant cell line, corresponding to a final dilution of 3×10^{-5}, or ~100 copies of the NsiI-Integrant allele. This was supplemented with an equimolar mix of the heterozygous c.1498A>G (p.Ile500Val) and c.1499T>C (p.Ile500Thr) gDNA samples and added in known amounts corresponding to final dilutions of 0 (no MYHRS DNA), 10^{-6} (0.02 ng of each MYHRS DNA), 3×10^{-6} (0.06 ng), 10^{-5} (0.2 ng), 3×10^{-5} (0.6 ng), and 10^{-4} (2 ng). The titration samples were all performed as technical duplicates and taken through the same protocol of NsiI selection and mutation enrichment as the sperm and blood samples (see below).

NsiI RED-PCR assay: Mutation selection and enrichment

An overview of the RED-PCR assay is presented in Figure S1. Individual biological samples (sperm or blood) were processed in triplicate: each technical replicate comprising 10 μg of gDNA spiked with 0.6 ng DNA from the NsiI-Integrant cell line (corresponding to a final dilution of 3×10^{-5} or ~100 mutant haploid genomes) was digested in a 100 μL volume reaction containing 20 U NsiI-HF and 20 U PstI-HF (both from New England BioLabs) for 2 h at 37°C, after which another 20 U of each enzyme was added and incubated for a further 2 h. NsiI mutant sequences generate a 1,332 bp PstI fragment, while the wild-type sequences are cleaved by NsiI into 2 fragments (488 and 844 bp). Each digest was loaded on a 1% agarose/TAE gel and flanked by a λ DNA/EcoRI ladder (Fermentas). Following electrophoresis at 4°C overnight, the ladder lanes were excised from the gel and stained with ethidium bromide for 20 min. Then, the 1,264 bp and 1,371 bp ladder bands were injected with gel loading dye under ultraviolet light and the gel was reconstructed. The narrow horizontal strip of each sample lane between the two marked ladder bands was excised and gel purified using a gel purification kit (Zymo). The purified DNA was eluted in 25 μL and was stored at −20°C until PCR amplification.

PCR amplification and library preparation

A first PCR (PCR1) was performed on the entire 25 μL gel purified sample using 0.8 U Q5 Hot Start High-Fidelity DNA polymerase (New England BioLabs), 1× Q5 buffer, 200 μM dNTPs, 0.5 μM of SMAD4-1F, and SMAD4-1R primers (278 bp product, Table S1) in a total reaction volume of 40 μL, and the following cycling conditions: 98°C (2 min), followed by 25 cycles of 98°C (10 s), 68°C (30 s), and 72°C (30 s), followed by a final extension at 72°C (2 min). The entire product was further enriched by re-digestion with 20 U NsiI-HF at 37°C for 1 h in a final volume of 100 μL. For each sample, an aliquot of 5 μL of the re-digested PCR1 material was used as template for a nested PCR amplification (PCR2) using 1 U DreamTaq DNA polymerase (ThermoFisher), 1× DreamTaq buffer, 0.1 μM dNTPs, and 0.1 μM each of the SMAD4_CS1-2F and SMAD4_CS1-2R primers (Table S1) in a final volume of 75 μL, generating a 208 bp product. Cycle conditions for PCR2 were as follows: 95°C for 5 min, followed by 20 cycles of 95°C (30 s), 64°C (30 s), and 72°C (30 s), followed by a final extension at 72°C (5 min). For the titration experiments and reproducibility assays (see below), PCR2 was performed in triplicate (3 independent PCR2) for each of the re-digested PCR1 samples; for the final assay assessing sperm and blood samples, PCR2 was performed only once. To construct an Illumina library, a unique 10 bp barcode was added to each PCR2 product in a separate 20 μL, 12 cycles PCR reaction, using 1 μL of a 1:100 dilution of the PCR2 products, 1× iProof High Fidelity Master Mix, and 0.4 μM of each PE1-CS1 and unique (sample-specific) PE2-BC-CS2, where the BC is a 10 bp (N_{10}) generic Access Array Barcode for Illumina sequencers (Fluidigm). The barcoded amplicons were pooled in near-equimolar ratio, and the final pool subjected to a further round of digestion with 1 U NsiI-HF for 1 h at 37°C. The digested product was separated on a 3% agarose/TAE gel, and the NsiI-resistant band was excised and purified using a gel purification kit (Zymo). The resulting library was quantified and assessed by TapeStation (Agilent) and diluted to ~10 ng/μL.

Ultra-deep massively parallel sequencing

Libraries were sequenced on a MiSeq platform (Illumina) with 2 × 151 paired-end reads using custom CS1-Seq primer (0.5 μM) for read 1, CS2-Seq primer for read 2, and RC-CS2 primer for the Indexing read.[42]

Bioinformatics analysis

The sequencing data were processed using Amplimap v.0.4.20[43] with parameters 'coverages pileups –njobs 10', as previously described.[42,44] Amplimap is a complete workflow for automating data analysis from targeted next-generation sequencing experiments and by default, and as used here, wraps BWA v.0.7.17-r1188, mpileup v.1.16.1, bedtools v.2.29.2, and GATK v.4.2.0.0, aligning reads to the human reference sequence GRCh38.p13. The fasta and GTF files for these assemblies were downloaded from Ensembl v.106 (https://www.ensembl.org/info/data/ftp/index.html). Additional information on the analysis of the sequencing data presented in this study is available at www.github.com/sjbush/smad4. Samples with read depth <1000 reads or with coverage of the NsiI-Integrant <250 reads (determined as <250 reads for *SMAD4* c.1494del) were excluded. For the titration and reproducibility experiments, the total read counts of the PCR2 technical triplicates were pooled together. To calculate the estimated mutation levels within the NsiI site (c.1494_1499ATGCAT) and the confidence intervals (CIs), we assumed that the counts of the mutations in the sequencing data (excluding the wild-type sequence) were multinomial and calculated the 95% confidence intervals (upper [UPR] and lower [LWR]). The method used for the CI of multinomial proportion is by Sison and Glaz,[45] implemented in R v.4.3.1 (https://www.R-project.org/) using the MultinomCI function of the DescTools v.0.99.54 package (https://cran.r-project.org/package=DescTools).

Functional assays

Cloning of SMAD4 coding sequence into mammalian expression vector

A 1,699 bp fragment containing the coding sequence of wild-type human *SMAD4* was amplified by PCR using Q5 HotStart High Fidelity DNA Polymerase master-mix (New England BioLabs) and control human cDNA as a template, according to manufacturer's recommendations (Table S1). The *SMAD4* fragment was cloned into the pCMV6-AC-HA mammalian expression vector (OriGene) using the Gibson method.[46] The successfully cloned construct was transformed into competent bacteria, positive colonies were cultured and vector DNA isolated using the GenElute Plasmid Miniprep Kit (Sigma). The sequence of the construct was verified by dideoxy-sequencing. *SMAD4* mutants were generated using a Q5 Site-Directed Mutagenesis Kit (New England BioLabs) according to manufacturer's instructions using the primers listed in Table S1 (designed according to https://nebasechanger.neb.com/), and identities of the mutants were verified by dideoxy-sequencing.

Dual-Glo luciferase assays

HEK293T cells (American Type Culture Collection) were cultured in Dulbecco Modified Eagle Medium (Gibco) supplemented with 10% fetal bovine serum (Gibco), 1% penicillin/streptomycin (Gibco), and 1% L-glutamine (Gibco) and grown at 37°C with 5% CO_2.

HEK293T cells (1 × 10^5) were seeded in 24-well culture plates and after 24 h were co-transfected with 5 ng Renilla internal control plasmid (Promega), 95 ng of pGL4.48[luc2P/SBE/Hygro] luciferase plasmid (Promega), and either 200 ng or 400 ng of pCMV6-AC-HA-SMAD4 wild-type or mutant plasmid, or empty pCMV6-AC-HA plasmid, using Lipofectamine LTX (Invitrogen) according to the manufacturer's protocol. After 48 h, dual luciferase reporter assays were performed using the Dual-Glo Luciferase Assay System (Promega) according to manufacturer's instructions and a Promega GloMax luminometer. The relative reporter activity was determined by normalizing firefly activity to Renilla activity.

Protein stability assays

HEK293T cells (4 × 10^4) were seeded in 6-well culture plates and incubated for 24 h at 37°C in a humidified atmosphere containing 5% CO_2. Cells were transfected with 1 μg of relevant pCMV6-AC-HA vectors (empty, wild-type *SMAD4* or each of the tested mutants), using the polyethylenimine (PEI) transfection reagent (Polysciences), according to the manufacturer's instructions. After 24 h, cells were treated for 2 h with 10 μg/mL of cycloheximide (CHX) (Sigma-Aldrich) and then lysed in radio-immune precipitation assay (RIPA) buffer (pH 8.0), supplemented with protease and phosphatase inhibitors (Sigma-Aldrich). Lysates were kept on ice for 30 min and then centrifuged at 16,000 × *g* for 20 min at 4°C. Supernatants were collected and their protein concentration was determined by Bradford assay (Bio-Rad Laboratories), using bovine serum albumin (BSA) (lot#: SLCL0258, Sigma-Aldrich) as a standard. Immunoblotting assays were performed as previously reported.[47] In brief, equal amounts of total proteins from cell lysates were resolved by sodium dodecyl sulfate (SDS)-polyacrylamide gel electrophoresis. Proteins were transferred to a nitrocellulose membrane using the Trans-Blot Turbo transfer system (Bio-Rad Laboratories). Blots were blocked with 5% non-fat milk powder in PBS containing 0.1% Tween 20 for 1 h and incubated at 4°C overnight with specific antibodies (3724S, rabbit polyclonal anti-HA [lot #: C29F4, dilution 1:1,000, Cell Signaling Technology] and sc-32233, mouse monoclonal anti-GAPDH [lot#: 6C5, dilution 1:1,000, Santa Cruz Biotechnology]). Primary and secondary horseradish peroxidase conjugated anti-mouse (31450, lot#: 093075I, dilution 1:3,000) and anti-rabbit (31460, lot#: WC32 0195, dilution 1:3,000, Invitrogen) antibodies were diluted in blocking solution. Immunoreactive proteins were detected by an enhanced chemiluminescence (ECL) detection kit (Thermo Fisher Scientific), according to the manufacturer's instructions. Densitometric analysis of protein bands was performed using NineAlliance UVITEC software

(UVITEC) and statistical analyses were carried out using a two-way ANOVA test in GraphPad Prism software v.8.4.3. The uncropped pictures of the western blotting membranes used for the densitometric analysis of SMAD4 protein levels are shown on Figure S2.

ERK phosphorylation assays

ERK phosphorylation assays were performed on transfected HEK293T cells (1 μg pCMV6-AC-HA vectors [empty, wild-type SMAD4, or each of the tested mutants]), seeded in 6-well plates the day before transfection (70%–80% confluence). Cells transfected with 1 μg pcDNA6.2/V5-DEST-LZTR1^{Ser247Asn} were used as positive control for ERK phosphorylation.[48] Cells were serum-starved for 16 h and subsequently stimulated with FBS (lot#: 2563330, 20% in DMEM, Thermo Fisher Scientific) or TGF-β1 (lot#: DCPU1123021, 20 ng/mL, Bio-Techne) for 5 and 15 min, or left unstimulated. Cells were lysed in RIPA buffer, samples were centrifuged at 16,000 × *g* for 20 min at 4°C, and protein concentration in supernatants was determined by Bradford assay. Immunoblotting analyses were performed as previously reported in protein stability assays using the following antibodies: sc-32233, mouse monoclonal anti-GAPDH (lot#: 6C5, dilution 1:1,000, Santa Cruz Biotechnology); R96025, mouse monoclonal anti-V5 (lot#: 2675745, dilution 1:1,000, Invitrogen); 3724S, rabbit polyclonal anti-HA (lot#: C29F4, dilution 1:1,000), 9102S, rabbit polyclonal anti-p44/42 MAPK (ERK1/2) (lot# 28: dilution 1:1,000) and 9106S, mouse monoclonal anti-phospho-p44/42 MAPK (ERK1/2) (lot# E10, dilution 1:2,000) (Cell Signaling Technology); horseradish peroxidase conjugated anti-mouse (31450, lot# 093075I, dilution 1:3,000) and anti-rabbit (31460, lot# WC320195, dilution 1:3,000) (Invitrogen). The uncropped pictures of the western blotting membranes used for the densitometric analysis of ERK1/2 phosphorylation levels performed following stimulation are shown in Figure S3 (FBS) and Figure S4 (TGF-β1).

Results

Parent-of-origin analysis of *de novo* MYHRS-causing *SMAD4* mutations

In order to determine the parent-of-origin of the *SMAD4* DNM in subjects with sporadic MYHRS, we collected DNA samples from 22 family trios and used a haplotyping strategy relying on the presence of an informative single-nucleotide polymorphism (SNP) in the vicinity of the DNM to distinguish the two parental alleles. An SNP was found to be informative when the proband was heterozygous and at least one of the parents was homozygous. Following the determination of the SNP informativeness in each family trio, the phase of the DNM in respect to the SNP was established and parent-of-origin assigned.[44,49] Among the 22 available family trios, 16 were found to have an informative SNP in the vicinity of the *SMAD4* MYHRS-causing DNM. These included 14 trios for which the SNP was identified via cloning and sequencing of ∼4 kb genomic sequence around the DNM site. Using Oxford Nanopore long-read sequencing on four family trios, informative SNPs located ∼6.5 kb away were identified for two further trios (Table S2). In all 16 informative families, the causative DNM was present on the paternally derived allele, a result statistically significant over the expected 80% paternal origin expected for DNMs (chi-square test, $p = 0.02$) and in line with previously studied PAE disorders (Table S2).[49]

De novo MYHRS-causing *SMAD4* mutations are associated with increased paternal age

To determine whether parents of MYHRS probands are significantly older than the population average, it is necessary to compare parental ages at the time of birth to a matched control population. Paternal age varies across different countries, demographic groups, local geography, and year on year. To mitigate for some of these aspects, we obtained age data from a series of 35 confirmed and well-characterized MYHRS probands born in the USA, and compared the age of their parents at time of birth to the year-matched USA population mean (1980–2020), obtained from National Vital Statistics System (NVSS) (see subjects, materials, and methods). This cohort includes individuals carrying three different MYHRS-causing *SMAD4* variants (12 with c.1486C>T encoding p.Arg496Cys [34%]; 19 with c.1498A>G encoding p.Ile500Val [54.5%]; and 4 with c.1499T>C encoding p.Ile500Thr [11.5%]). On average fathers of MYHRS probands were 37.3 years old and showed a 6.3 (±1.1 SEM) year excess compared to the year-matched average age of fatherhood in the USA, a difference that was statistically significant (one tailed t-test, $p = 1.68 \times 10^{-6}$) and in line with the age excesses previously reported for other canonical PAE disorders (Table 1; Figure 1).

Because MYHRS-causing *SMAD4* DNMs show an exclusive paternal origin, analysis of maternal ages is not anticipated to be relevant. Nevertheless, given that maternal and paternal ages are correlated variables,[54,55] we performed a similar analysis for maternal ages and found a 4.13 (±0.79 SEM) years average maternal age excess in the USA cohort (Figure S5A).

As we were not able to perform parent-of-origin studies for the USA cohort, we also examined the age of fathers for the European (EU) family trios in which we had demonstrated the exclusive paternal origin of the MYHRS-causing DNMs. We had access to paternal age data for 17 EU families, among which 14 had proven paternal origin. Age data for this small cohort was compared to year-matched birth data of the UK average population and showed a mean paternal age (±SD) of 37.0 ± 8.9 years, which is significantly older than the year-matched UK population average (one tailed t test, *p* value = 0.023) and represents a comparable paternal age excess (mean ± SEM = 5.1 ± 2.3 years) to that observed for the whole USA cohort (Figure S5B).

Table 1. **Overview of the epidemiological paternal age effect (PAE) for MYHRS and other known PAE mutations**

Syndrome	Mutated gene	Mean paternal age ± SD (years)	Number of cases	Paternal age excess	Reference
Myhre syndrome	*SMAD4*	37.3 ± 6.7	35	6.3 years	this study
Apert syndrome	*FGFR2*	33.0 ± 6	51	2.3 years	Moloney et al.[50]
Crouzon/Pfeiffer syndromes	*FGFR2*	34.5 ± 7.7	30	4.1 years	Glaser et al.[51]
Achondroplasia	*FGFR3*	35.6 ± 7.2	39	7.1 years	Wilkin et al.[52]
Muenke syndrome	*FGFR3*	34.7 ± 7.7	19	4.1 years	Rannan-Eliya et al.[53]
Noonan syndrome	*PTPN11*	35.6	15	6.1 years	Tartaglia et al.[49]

MYHRS-associated Ile500 variants are prevalent in sperm of men of different ages

In order to determine whether the MYHRS-causing mutations are subject to positive selection in human testes, we sought to quantify the levels of spontaneous *SMAD4* mutations directly in sperm of men of different ages. We anticipated that if these mutations are clonally expanding in human testes, they would progressively become enriched in aging testes to reach detectable levels in sperm and increase in prevalence with donor's age. We adapted a previously published protocol for ultra-rare mutation detection based on mutation enrichment using a restriction enzyme digestion and PCR amplification (RED-PCR) approach, followed by ultra-deep sequencing of the region of interest (Figures 2 and S1).[14] Using the restriction enzyme NsiI on DNA extracted from sperm and blood samples, mutant sequences within the NsiI site located at position c.1494_1499ATGCAT (cDNA GenBank: NM_005359.6 [NP_005350.1]) can be enriched. Of note, while the NsiI digestion overlaps with two of the most common MYHRS-causing *SMAD4* variants (c.1498A>G [p.Ile500Val] and c.1499T>C [p.Ile500Thr]), this strategy enriches equally all 18 possible nucleotide substitutions within the 6-bp NsiI site (affecting codons Leu498, Cys499, and Ile500) (Figure 2A).

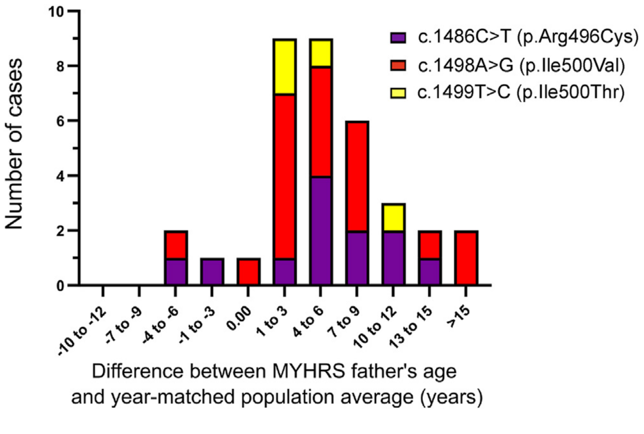

Figure 1. Paternal age effect for *de novo* MYHRS-causing *SMAD4* mutations
Distribution of the observed difference between the ages of fathers ($n = 35$) of MYHRS-affected individuals and year-matched USA population average (pooled in 3-year bins). The shift to the right side of the figure illustrates the paternal age excess. The specific *SMAD4* mutation identified in the proband is denoted according to the color chart indicating the MYHRS variant and predicted amino acid change. The maternal age effect for these families is shown in Figure S5A.

To assess the capability and sensitivity of the assay to detect mutations at ultra-low levels (down to the range of 10^{-5} to 10^{-6}, based on the expected rarity on these mutations and the low birth prevalence of MYHRS), we performed a titration-reconstruction experiment using two technical replicates each containing 10 μg of control gDNA extracted from blood (equivalent to ~3.3×10^6 copies of the haploid genome) supplemented with a dilution series of equimolar gDNA from two individuals heterozygous for the *SMAD4* c.1498A>G (p.Ile500Val) (MYHRS 1) or c.1499T>C (p.Ile500Thr) (MYHRS 2) variants (range of input mutant molecules from ~3 [dilution: 1×10^{-6}] to ~333 [dilution: 1×10^{-4}]). To obtain an absolute quantification of mutation levels following NsiI digestion, we used a CRISPR-Cas9 genome modification approach to engineer an NsiI-resistant clone and obtained a clone containing a 17 bp heterozygous deletion in *SMAD4* corresponding to cDNA position c.1478_1494del (see subjects, materials, and methods). As the final base of the deletion encompasses the first base of the NsiI cut site (position c.1494A), the mutant allele is resistant to NsiI digestion (c.1494_1499<u>A</u>TGCAT) (Figures 2A and S1A). Conveniently, this NsiI-Integrant clone has a unique genomic signature, making it well suited to be used as a molecular spike to quantify absolute mutation levels across the NsiI site. For accurate quantification of the dilution series, all titration samples were spiked with 100 mutant copies (dilution equivalent to 3×10^{-5}) heterozygous gDNA from the NsiI-Integrant clone. Figure 2B shows a robust correlation ($R_s = 0.98$–0.99) between the levels of input for each of the two MYHRS gDNA samples and the mutation levels estimated by massively parallel sequencing, following two rounds of NsiI digestion and PCR amplification, indicating a good sensitivity of the assay to measure MYHRS mutation levels down to levels as low as ~1×10^{-6} (Figure 2B; Table S3).

To assess the reproducibility of the assay across biological and technical replicates, we used the same strategy to measure all 18 single nucleotide substitutions within the NsiI restriction enzyme site in technical triplicates of 12 sperm samples from four different individuals taken at

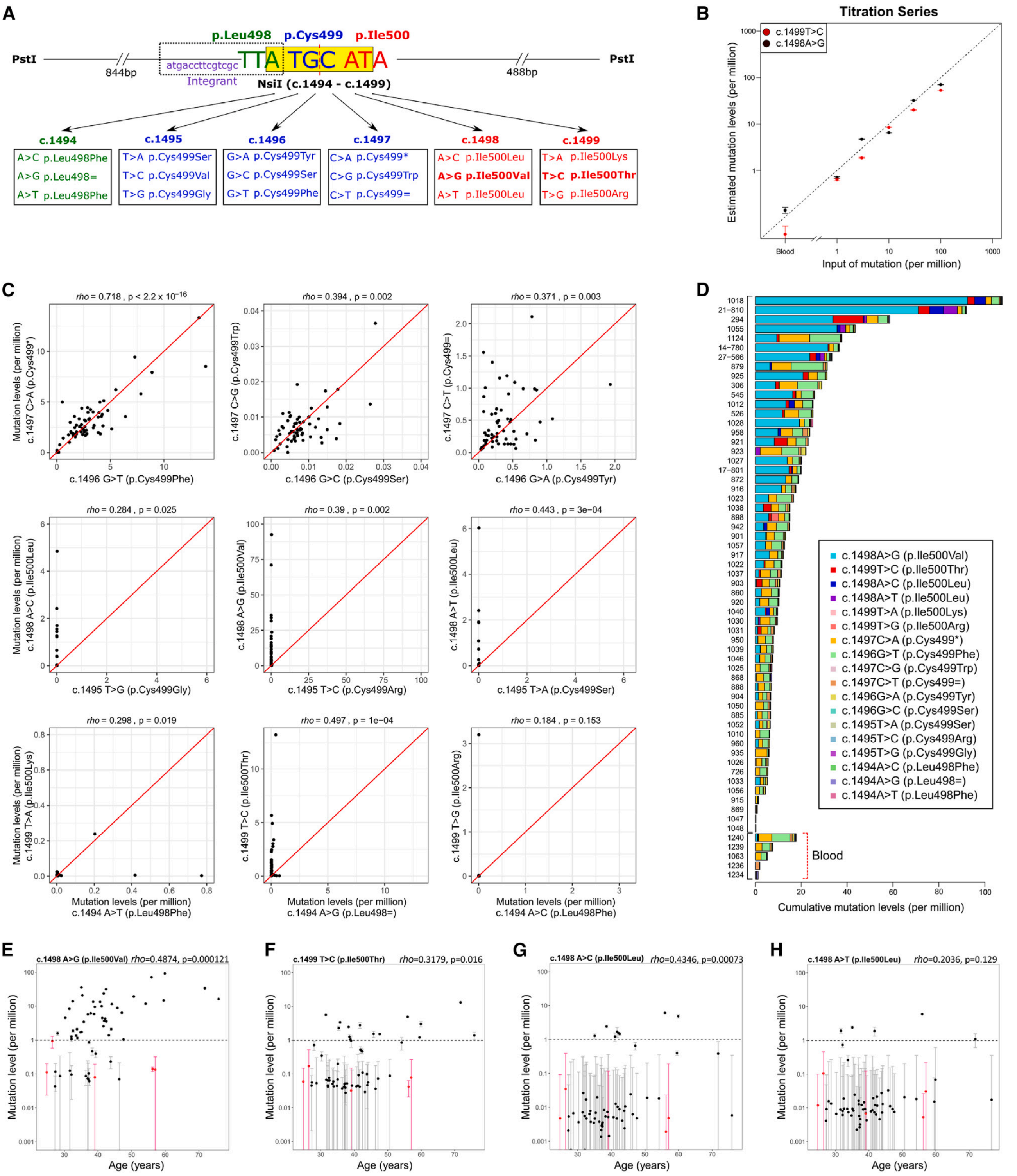

Figure 2. NsiI restriction enzyme digestion (RED)-PCR assay for enrichment of spontaneous MYHRS-associated *SMAD4* mutations
(A) *SMAD4* sequence context around codon 500 (red), which encompasses an NsiI restriction site (yellow box—note the palindromic nature of the site, indicated by the dotted red line). PstI digestion releases a 1,332 bp gDNAfragment and wild-type sequences are also digested with NsiI to generate two smaller fragments (844 and 488 bp) (Figure S1). All nucleotide substitutions (and corresponding amino acid changes) resistant to NsiI digestion are indicated and are color coded according to the amino acid involved. The two known MYHRS-causing mutations that can be enriched by this strategy are indicated in bold. The CRISPR-edited Integrant clone, a 17 bp (c.1478_1494del) heterozygous deletion (in purple within the dotted rectangle), is also resistant to NsiI digestion.
(B) Mutation levels estimated in a titration-reconstruction assay with serial dilutions of two gDNA from MYHRS-affected individuals with the p.Ile500Thr (red) or p.Ile500Val (black) variant (as indicated on the figure) mixed with blood carrier gDNA and the Integrant DNA. Values plotted are means of two technical replicates and error bars represent 95% binomial confidence intervals (CIs) (see Table S3).

(legend continued on next page)

different time points (Figure S6; Table S4). For donors 21 and 27, we had access to DNA extracted from two independent ejaculates collected within three days of each other (biological replicates). For donors 14 and 17, DNA from four ejaculates were available, which included two independent biological samples taken three days apart and a further two biological samples taken three years later. We obtained consistent mutation level estimates across the 18 substitutions of the Nsi-site both in technical replicates and from different samples of the same individual (even for those collected three years apart), indicating that analysis of single sperm samples at one time point accurately reflects mutation prevalence in the individual (Figure S6). We next quantified mutation levels for all 18 substitutions across the NsiI site (c.1494_1499; Leu498, Cys499, Ile500) in 5 blood and 57 sperm samples from healthy individuals without family history of MYHRS, which were run as technical triplicates (Figures 2C–2E and S7; Table S5). To analyze the data agnostically, we considered the mutation levels for substitutions at each of the codon encompassing the NsiI site in turn and exploited the fact that the recognition site of the enzyme has a palindromic structure (Figure 2A). The mutation levels at c.1494A, which encodes the last position of the Leu498 codon, did not differ between sperm and blood samples and were not present above background ($\sim 10^{-6}$) in any sample (Figure S7; Tables S5 and S6). For substitutions affecting the nucleotides encompassing Cys499 (c.1495_1497), two substitutions (c.1496G>T [p.Cys499Phe] and c.1497C>A [p.Cys499*]) had mutation levels above background in the majority of samples; however, the mutation levels were similar in sperm and blood samples and did not correlate with donor age ($R_S = 0.125$ for c.1496G>T and $R_S = -0.006$ for c.1497C>A). Rather, the levels of c.1496G>T strongly correlated with the c.1497C>A levels within the same sample ($R_S = 0.718$, $p < 2.2 \times 10^{-16}$) (Figures 2C and S7; Tables S5 and S6). Given that these G>T/C>A transversions are present in all samples regardless of type or age and affect reciprocal positions within the palindromic NsiI site, they are likely to result from a technical artifact. Consistent with this interpretation, G>T transversion is a known heat-induced mutational signature associated with oxidative stress arising during DNA preparation or long-term storage.[56] The levels of the other seven substitutions affecting the Cys499 codon were at or below 10^{-6} (limit of detection) for all samples, both blood and sperm (Figure S7; Tables S5 and S6). In contrast, at the last 2 positions of the NsiI site, encoding the Ile500 codon, the c.1498A>G (p.Ile500Val) variant was present above background levels in 42/57 (73%) sperm samples, but in none of the blood samples, and showed a significant and positive correlation between mutation levels and donor's age ($R_S = 0.487$) (Figure 2E). The mean mutation level in sperm (the mean age of the sperm donors was 40.71 years [range: 27.22–75.86]) was 9.33 per million, with a maximum of 92.5 per million in a sample from a 59.9-year-old man. Likewise, the level of the c.1499T>C (p.Ile500Thr) MYHRS-causing variant was detected above background levels in 14/57 (25%) sperm samples, but in none of the blood samples, with a positive (although weaker than for p.Ile500Val) correlation between mutation levels and donor age ($R_S = 0.318$) (Figure 2F). The mean mutation levels measured in sperm across the 57 sperm samples was 0.9 per million, with a maximum of 13.2 per million in a sample from a man aged 71.8 years. Unlike the correlation observed between the c.1496G>T and its reciprocal c.1497C>A, the cognate positions within the NsiI palindrome to the MYHRS variants (i.e., c.1495T>C [p.Cys499Arg] is reciprocal to c.1498A>G [p.Ile500Val] and c.1494A>G [p.Leu498=] is reciprocal to c.1499T>C [p.Ile500Thr]) did not show elevated mutation levels (Figures 2C and S7). At the Ile500 codon, there are two alternative DNA substitutions resulting in an isoleucine-to-leucine change, c.1498A>C and c.1498A>T. While the p.Ile500Leu has not previously been reported as a MYHRS recurrent mutation or in genomic variation databases, c.1498A>C was present above the assay limit of detection (10^{-6}) in sperm samples from eight men and positively correlated with donor age ($R_S = 0.435$), while c.1498A>T was found at elevated levels in samples from five individuals ($R_S = 0.204$) (Figures 2G and 2H). Neither of these substitutions were present above background levels in any of the blood samples or at their reciprocal positions in the

(C) Comparison of the mutation levels (per million) in sperm samples for the equivalent (i.e., reciprocal) substitutions within the palindromic NsiI site (ATG|CAT)—note that for the top row (and in particular the top left panel), the c.1497C substitution levels within individual samples correlate significantly with the levels of their cognate c.1496G change. This pattern is best explained by the DNA substitutions occurring through a passive process, such as oxidative damage. By contrast for the other substitutions (middle and bottom rows), the relationships between equivalent substitutions are different, with mutation levels of reciprocal/cognate substitutions not correlating with one another. Note the variable range of the axes that have been adjusted to reflect the levels of the highest measurements.

(D) Cumulative mutation levels for the 18 different substitutions enriched by the NsiI RED-PCR assay (see color chart) observed in sperm (top) and blood (bottom) samples. Mutations encoding the p.Ile500Val, p.Ileu500Thr, and p.Ileu500Leu changes dominate the landscape, while the c.1496C>A and its cognate c.1497G>T variant are found in similar proportions. Individual plots are presented in Figure S7.

(E–H) Mutation levels of four apparently selected variants at Ile500 (c.1498A>G [p.Ile500Val], c.1499T>C [p.Ile500Thr], and c.1498A>C and A>T which both encode p.Ile500Leu) plotted against the age of the donors on a log10 scale. The dotted line represents the 10^{-6} limit of detection of the RED-PCR assay. Blood (red) and sperm (black) samples are plotted as the mean of three independent technical replicates and their 95% binomial CI.

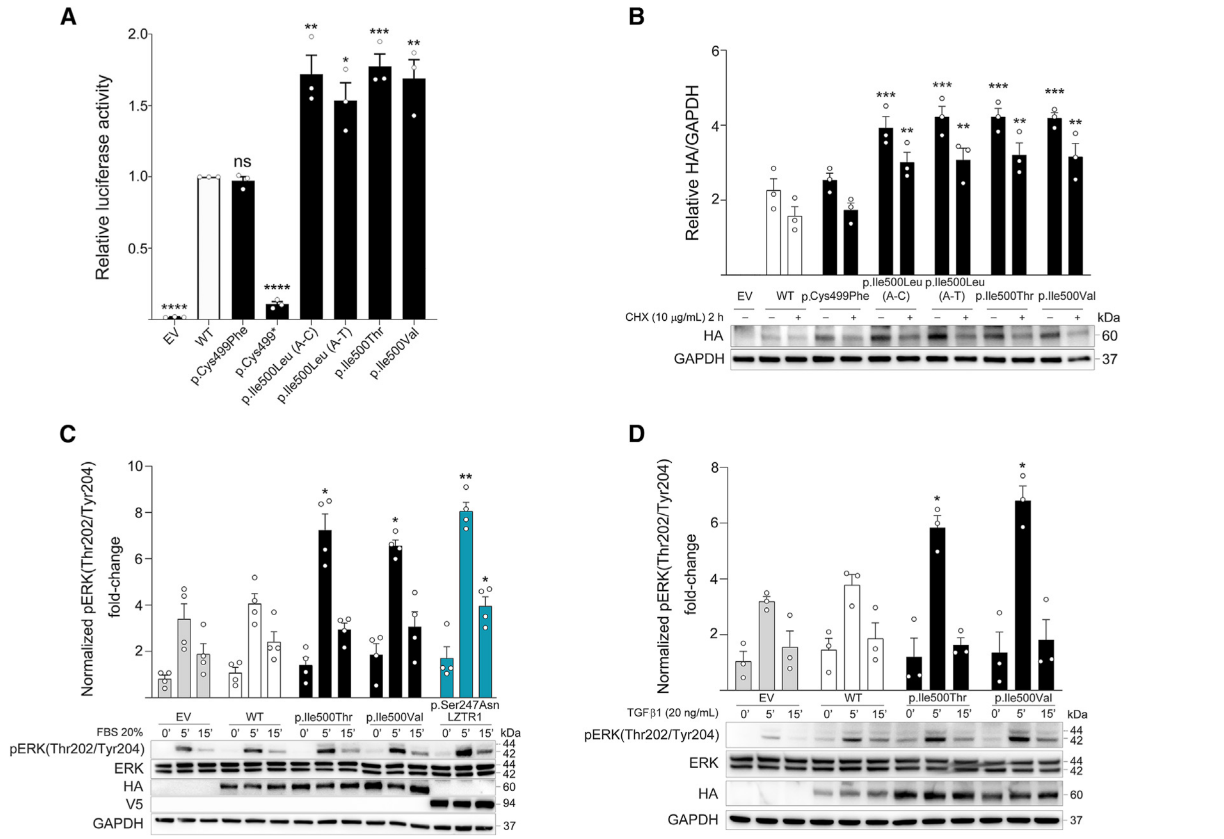

Figure 3. Functional characterization of the sperm-enriched *SMAD4* variants

(A) Transcriptional activity (assessed by luciferase assay using SBE3 binding site in HEK293T cells) of wild-type (WT) and enriched SMAD4 protein mutants, as indicated on the figure. The graph bars represent mean ± SEM and values of 3 individual replicates are indicated as white circles.

(B) Protein levels and stability of SMAD4 mutants. Immunoblot analysis shows WT and variant HA-tagged SMAD4 protein levels in transfected HEK293T cells, basally and after cycloheximide (CHX) treatment. GAPDH was used as loading control. Representative blots (below) and mean ± SEM densitometry values (above) of three independent experiments are shown. Asterisks indicate statistically significant differences compared to the WT SMAD4 (***$p \leq 0.001$; **$p \leq 0.05$; two-way ANOVA followed by Dunnett's multiple comparison test).

(C and D) Serum-stimulated phospho-ERK (pERK) assay. Representative blots (below) and graphs reporting mean ± SEM densitometry values (above) of at least three independent experiments are shown. HEK293T cells were transiently transfected with the indicated HA-tagged *SMAD4* or V5-tagged *LZTR1* construct, serum starved (16 h), and stimulated with FBS (C) or TGF-β1 (D), in time-course experiments or left untreated. Equal amounts of total proteins from each cell lysate were resolved on 10% polyacrylamide gels. Asterisks indicate statistically significant differences in the phosphorylation levels compared to cells transfected with empty vector at the corresponding experimental points (**$p < 0.01$; *$p < 0.05$; two-way ANOVA followed by Dunnett's multiple comparison test).

NsiI site (Figure 2C). For the other two substitutions affecting the Ile500 codon, c.1499T>G (p.Ile500Arg) was observed above background in a single sperm sample at a level of 3.2 per million, while c.1499T>A (p.Ile500-Lys) was below background levels in all sperm and blood samples (Figures 2C and S7). Taken together, these data indicate that the two previously reported MYHRS-causing *SMAD4* mutations are detected at elevated levels in the sperm of most men and the levels show an increase with donor's age. This behavior is consistent with clonal selection driven by the encoded protein changes rather than hypermutability of the specific DNA substitutions. Moreover, our data suggest that the p.Ile500Leu variants (encoded by two different DNA substitutions) may also be under positive selection in aging testes.

Functional impact of positively selected *SMAD4* missense variants

To refine our interpretation of the significance of the sperm-enriched MYHRS-associated *SMAD4* variants (p.Ile500Val, p.Ile500Thr, and p.Ileu500Leu), which exhibit a signature of selection, and those detected at levels above background (p.Cys499Phe and p.Cys499*), we performed *in vitro* assays to evaluate their functional behavior. First, we used a dual luciferase assay to establish the impact of the different SMAD4 mutants on the expression of a TGF-β-dependent reporter target construct. Wild-type and mutant *SMAD4* cDNAs were cloned into the p.CMV6-HA-CA mammalian expression vector and co-transfected into HEK293T cells, along with a firefly luciferase reporter gene under the control of three copies of a SMAD-binding element (SBE3) (Figure 3A), as previously described.[29] The

p.Ile500Val and p.Ile500Thr mutant proteins both showed a small but significant increase in luciferase activity compared to the wild-type construct (1.69 RLU and 1.77 RLU, respectively). Similarly, both constructs encoding the p.Ile500Leu SMAD4 mutants, which had not previously been associated with MYHRS, also resulted in a significant increase of luciferase expression (1.72 RLU and 1.54 RLU for the c.1498A>C and A>T changes, respectively). By contrast, the p.Cys499Phe mutant showed no significant difference in luciferase expression compared to the wild-type protein, and the p.Cys499* construct resulted in complete loss of luciferase expression—at a level similar to the empty vector—suggesting that the truncated protein is non-functional (Figure 3A).

We then assessed the effects of the *SMAD4* selected variants at codon Ile500 on protein stability (Figure 3B). Because Ile500 lies in close proximity to Lys519, a known site of SMAD4 ubiquitination, it has been previously proposed that the pathophysiological mechanism of MYHRS involves an impaired/defective protein ubiquitination affecting protein stability and/or function.[22] Indeed, Le Goff et al. showed increased levels of SMAD4 protein and reduced SMAD4 ubiquitination in fibroblasts from individuals heterozygous for the p.Ile500Thr variant compared to control individuals.[21] Based on these considerations, we performed a protein stability assay for the panel of selected mutants and found that proteins carrying the MYHRS-associated p.Ile500Val, p.Ile500Thr, and p.Ile500Leu (both permutations) amino acid substitutions were significantly more stable than wild-type SMAD4—both basally and after cycloheximide (CHX) treatment. No significant difference of protein stability was observed between the four selected mutants. By contrast, the p.Cys499Phe mutant protein exhibited protein levels and a stability profile indistinguishable to those observed for wild-type SMAD4 (Figure 3B). As the truncated p.Cys499* mutant does not produce a full-length protein, its function was not assessed in this assay. Taken together, these results show that the four *SMAD4* variants at codon Ile500 found to be enriched in sperm exhibit similar transcriptional activity *in vitro*, likely resulting from an increased protein stability, with the two substitutions encoding p.Ile500Leu mutants acting similarly to the p.Ile500Val and p.Ile500Thr MYHRS-causing variants. By contrast, the p.Cys499Phe and p.Cys499* mutant proteins did not increase the SMAD4 transcriptional activity, with the p.Cys499Phe mutant being indistinguishable to the wild-type protein and the truncated p.Cys499* producing a non-functional protein. These results further support our interpretation that c.1496G>T (p.Cys499Phe) and c.1497C>A (p.Cys499*), despite being observed at elevated levels in semen samples, are technical artifacts likely resulting from heat stress occurring during DNA extraction.

While SMAD4 is a key effector of the TGF-β/BMP/activin signaling pathway, all the evidence collected so far suggests that clonal selection of spermatogonial stem cells is mediated through upregulation of the RAS-MAPK signaling cascade. Hence, we asked whether the sperm-enriched SMAD4 mutants were able to perturb the RAS-MAPK signal flow *in vitro*. To this end, we first investigated the levels of phosphorylated ERK (pERK), the last tier of the MAPK pathway, in HEK293T cells transiently transfected to express wild-type or mutant *SMAD4* constructs, in time-course experiments upon stimulation with a cocktail of growth factors (20% FBS).[57] A dominant-negative *LZTR1* mutant construct, previously shown to upregulate ERK phosphorylation in stimulated conditions, was used as a positive control in the assay.[48] As shown in Figure 3C, the positively selected Ile500 mutants promoted a stimulus-dependent upregulation of ERK phosphorylation, in a manner similar to the dominant Noonan syndrome-associated LZTR1 mutant. To further delineate the pathway involved in SMAD4 activity, we performed the same pERK assay where FBS induction was replaced by TGF-β1 stimulation (Figure 3D). In this condition, overexpression of wild-type *SMAD4* did not result in a significant perturbation of pERK levels, neither basally nor following growth factor stimulation. By contrast, both p.Ile500Val and p.Ile500Thr SMAD4 mutants promoted a significant stimulus-dependent increase of ERK phosphorylation. These *in vitro* findings suggest that the MYHRS SMAD4 mutants act not only through a GoF mechanism within the canonical TGF-β signaling pathway, but may also confer a "neomorphic" function to SMAD4, by providing the mutant proteins with the ability to modulate a crosstalk with the RAS-MAPK signaling cascade.

Discussion

In this study, we explored the possibility that MYHRS-causing *SMAD4* mutations are under positive selection in the adult male germline. To this end, we asked whether MYHRS presentation's features fulfill the three criteria previously observed for *bona fide* PAE disorders. First, the analysis of the parental origin of MYHRS-causing variants in 16 informative family trios via haplotype phasing showed that the DNMs arose on the paternally derived allele in all cases, demonstrating the exclusive paternal origin of the *SMAD4*-causing mutations. Using parental age data of a well-characterized MYHRS cohort, we then documented that men who fathered a child with a pathogenic *SMAD4* DNM were on average 6.3 years older when compared to year-matched population average, demonstrating a statistically significant epidemiological paternal age effect for MYHRS. Finally, we developed an ultra-sensitive sperm assay to quantify the levels at which spontaneous MYHRS-causing *SMAD4* variants are observed in sperm samples and showed that MYHRS-associated *SMAD4* variants at codon 500 are found at elevated level in sperm of most men and exhibit a strong and significant positive correlation with donor age, indicative of a high apparent germline mutation rate. Taken together, these findings strongly support a model in which spontaneous

MYHRS-associated variants become progressively enriched in the testes of aging men via a mechanism of clonal selection of mutant SSCs over the course of time. Importantly, this study provides compelling evidence that selfish spermatogonial selection can be mediated by genes located outside the canonical RAS-MAPK signaling pathway.

We showed that the RED-PCR assay allows unbiased quantification of all spontaneous mutations within six base-pairs (within the NsiI site) of *SMAD4* in sperm of men of different ages. This approach has several advantages that have allowed us not only to detect selfish behaviors of specific mutations but also to anticipate the relative prevalence of pathogenic DNMs in the population. For example, we showed that there were 2.86 times more sperm samples carrying detectable levels of the pathogenic p.Ile500Val than the p.Ile500Thr variant. These data are consistent with the observation that p.Ile500Val is at least twice or three times as frequent as p.Ile500Thr in MYHRS.[58,59] The mean mutation level in sperm across the sperm donors cohort for p.Ile500Val (~1:100,000) was also considerably higher than that for p.Ile500Thr (~1:1,000,000), suggesting that the clonal selection of the former may be stronger. Our *in vitro* functional assays did not show a differential impact of the two missense changes on SMAD4 transcriptional activity or protein stability, suggesting that additional aspects of SMAD4 function, that have not been assessed here, likely contribute to the differential clonal selection driven by the p.Ile500Val and p.Ile500Thr variants. Our data also identified two other nucleotide substitutions, both encoding a p.Ile500Leu amino acid change, as being enriched in sperm. Based on similarity of amino acid physico-chemical properties with the other known pathogenic MYHRS-associated variants, we reasoned that the p.Ile500Leu mutants could also be pathogenic, a proposal that was supported by our *in vitro* functional assays that demonstrated the p.Ile500Leu SMAD4 variants behaved indistinguishably to p.Ile500Val and p.Ile500Thr. Interestingly, the p.Ile500Leu variant has recently been reported as likely causative in an individual with mild features of MYHRS[60] and as a *de novo* variant of uncertain significance in a proband diagnosed with type 2 congenital microcephaly 2 (MCPH2 [MIM: 604317]) who carried inherited compound heterozygous variants in *WDR62* (MIM: 613583).[61] Our findings of enrichment of these variants in sperm and their *in vitro* activity further support the likely pathogenicity of p.Ile500Leu. Of note, it is striking that despite being pathogenic, all MYHRS-causing variants encode conservative amino acid changes (for example, the "Grantham difference" score for Ile>Val is 29 and for Ile>Leu is 5)[62] and are likely to confer subtle GoF properties to mutant proteins. Given that Ile500 lies in close proximity to Lys519, a known site of ubiquitination, our protein stability assays, confirming the increased stability of the SMAD4 mutants over wild-type protein, are consistent with the previous hypothesis that subtle protein structural rearrangements caused by MYHRS mutations may affect ubiquitination at this site.[21–23]

As a rare disorder with a narrow mutational target within *SMAD4*, it is difficult to obtain an accurate estimate of the population prevalence of MYHRS. Based on our data, the average mutation levels quantified in sperm for the most common MYHRS variant (p.Ile500Val) were ~1:100,000 across a cohort of sperm donors aged on average 40.7 years (median age = 38.3 years [range: 27–76 years]), which is significantly older than the mean age of fatherhood. These levels measured in sperm are in line with those previously observed for other PAE disorders—for example, the *FGFR2*-causing Apert syndrome c.755C>G (GenBank: NM_000141.5) (p.Ser252Trp) change (estimated to account for ~1:100,000 births) was quantified at an average level of ~1:40,000 in sperm of a cohort of men aged on average 39.1 years (median age = 37.0 [range: 24–73 years]).[8] While the mutation levels measured in sperm demonstrate that MYHRS-causing variants are present more abundantly than other non-pathogenic substitutions in the male germline, they may not reflect directly the birth rate of this rare disorder given that some affected pregnancies may not reach term or alternatively, MYHRS may be underdiagnosed. We note the presence of 7 individuals with *SMAD4* c.1486C>T (p.Arg496Cys) and one individual carrying the c.1498A>G (p.Ile500Val) change in the gnomAD population database (gnomAD v.4.1.0), suggesting that some of the *SMAD4* pathogenic variants are not fully penetrant.

The identification of clonal selection of MYHRS-associated *SMAD4* variants in the male germline represents an unanticipated finding as components of the TGF-β/BMP/activin signaling have not been associated previously with paternal age effects or selfish selection. Yet, this is intriguing because disorders associated with mutations in genes of the TGF-β/BMP/activin pathway, such as Marfan syndrome (MIM: 154700), Loeys-Dietz syndrome (MIM: 610168), Shprintzen-Goldberg (MIM: 182212), or fibrodysplasia ossificans progressive (FOP; MIM: 135100) share some common features with known PAE disorders.[54,63]

SMAD4, also known as co-SMAD, operates at the core of the TGF-β/BMP/activin superfamily where it acts as an obligatory component of the signaling cascade, forming transcriptionally active heterotrimeric complexes with R-SMADs that are able to translocate to the nucleus and control expression of target genes.[27,28,64] *SMAD4* is expressed at high levels in several testicular cell types, including somatic cells (Sertoli and Leydig cells) and germ cells. Tissue-specific knock-out murine models have demonstrated an essential role for *Smad4* in Sertoli cells[65] but its precise role in adult spermatogonial stem cells remain poorly characterised.[66] Recent single-cell transcriptomics data confirmed that *SMAD4* is expressed in undifferentiated human spermatogonial stem cells[67] and that members of the TGF-β superfamily (including activins, BMPs, and TGF-βs) are key regulators of testis development and play a key role in spermatogonial homeostasis.[35,68,69]

Specifically, BMPs and activins have been reported to promote specification, survival, and proliferation of murine germ cells and are required for maintenance of adult spermatogenesis.[68,70–74] By contrast, TGF-β signaling has been documented to have a key inhibitory role during testis development by promoting germ cell quiescence.[75] Further work is required to demonstrate or rule out a direct relevance of the "canonical" TGF-β/BMP signaling mediated by R-SMAD-SMAD4 complexes in mutant germ cells carrying MYHRS-causing variants. However, it is important to consider that signal transduction elicited in response to growth factors of the TGF-β superfamily can also involve the activation of "non-canonical" pathways.[76] In this context, SMADs likely act as key nodes mediating cross-talk between major signaling pathways and orchestrate coherent cell decisions during development and/or homeostasis.

With this in mind, it is relevant to consider our finding showing that the levels of phosphorylated ERK are increased in response to activation by the SMAD4 Ile500 sperm-enriched mutants compared to the wild-type protein. This suggests that the functional impact of the SMAD4 mutants may also (at least in part) be mediated via increased activation of the RAS-MAPK pathway. This "neomorphic" function of the MYHRS-associated *SMAD4* mutations suggests a potential cross-talk between the TGF-β/BMP and RAS-MAPK signaling pathways. While experimental evidence of a direct functional interaction between SMADs and the MAPK cascade is limited, previous studies have indirectly implicated a potential cross-talk through shared regulators or effectors.[28] For example, TGF-β has been reported to modulate the amplitude and duration of MAPK activation with slow kinetics, suggesting a contribution associated with SMAD-dependent transcription responses; however, evidence also exists for rapid activation of ERK, the downstream tier of the MAPK cascade, in response to TGF-β stimulation, suggesting independence from transcription.[77] Moreover, the SMAD4 Thr277 residue in the linker region of SMAD4 is phosphorylated by MAPK-ERK in response to FGF and TGF-β, which facilitates its transcriptional activity and stability.[78] Likewise, the protein phosphatase WIP1 antagonizes FGF-induced ERK-mediated phosphorylation of SMAD4.[79,80] While the cross-talk between the TGF-β/BMP and MAPK signaling cascades is likely complex and context dependent,[76,81,82] the short-term enhanced MAPK signaling associated with the expression of MYHRS-causing SMAD4 mutants that we observed following serum and TGF-β1 stimulation suggests that positive selection of MYHRS variants in testes may be driven via upregulation of the MAPK signaling cascade. Although dedicated efforts are required to dissect the mechanisms of this modulatory role, these findings further emphasize the predominant role of the RAS-MAPK signaling upregulation in clonal selection in human SSCs.

Taken together, we have provided compelling evidence that MYHRS-associated DNMs in *SMAD4* are under positive selection in the male germline. As SMAD4 is an integral part of the TGF-β/BMP/activin signaling pathway, its association with selfish spermatogonial selection highlights the important role of this signaling cascade in controlling homeostasis in human testes. Our approach also demonstrates that screening of sperm or human testes provides a means to identify functional/pathogenic mutations and predict their pattern of occurrence in the population. Our findings have important implications for genetic counseling and prenatal testing, and also for public health advice more generally, as the average age of fatherhood continues to increase widely across the developed world.

Data and code availability

The accession number for the sequencing datasets generated during this study is European Nucleotide Archive: PRJEB 64495; http://www.ebi.ac.uk/ena/data/view/PRJEB64495.

Supplemental information

Supplemental information can be found online at https://doi.org/10.1016/j.ajhg.2024.07.006.

Acknowledgments

We acknowledge the support of the Myhre Syndrome Foundation, the European Reference Network ITHACA, and the WIMM core funding from the Medical Research Council (MRC) through the WIMM Strategic Alliance (G0902418 and 492 MC_UU_12025). We thank Tim Rostron and the members of the Oxford Genomic Medicine laboratory for technical support. This work was primarily supported by grants from the Wellcome Trust (219476/Z/19/Z to A.G.), the National Institute for Health Research (NIHR) Oxford Biomedical Research Centre Award (BRC 593413 to A.G. and S.R.F.T.), and Ministero della Salute (Current Research Funds and PNRR-MR1-2022-12376811 to M.T.) Ministero della Ricerca (FOE_2020 to M.T.), and the Telethon Undiagnosed Diseases Program GSP15001 (to N.B.-P.). R.S.T. is the recipient of a Sir David Weatherall Graduate Scholarship (Oxford). The funders had no role in study design, data collection and analysis, decision to publish, or preparation of the manuscript.

Author contributions

Conceptualization, A.O.M.W., M.T., and A.G. Experiments, K.A.W., R.S.T., M.M., V. Caputo, D.W.M., V. Cordeddu, A.T., and C.M. Age data collection, E.R.S. and A.E.L. Data analysis, K.A.W., R.S.T., M.M., S.J.B., E.R.S., E.G., A.E.L., M.T., and A.G. Provision of patient samples, G.B.F., F.B., P.G., D. M., K.S., N.B.-P., E.T., and V.C.-D. Writing, K.A.W., M.T., and A.G. Figures, K.A.W., M.M., E.R.S., S.J.B., D.W.M., E.G., M.T., and A.G. Supervision, A.O.M.W., A.E.L., V.C.-D., S.R.F.T., M.T., and A.G. Project administration, M.T. and A.G. Funding acquisition, M.T. and A.G. All authors have read and agreed to the submitted version of the manuscript.

Declaration of interests

The authors declare no competing interests.

Received: June 7, 2024
Accepted: July 3, 2024
Published: August 7, 2024

Web resources

COSMIC, https://cancer.sanger.ac.uk/cosmic

dbSNP, https://www.ncbi.nlm.nih.gov/snp

England and Wales population data, https://www.ons.gov.uk/peoplepopulationandcommunity/birthsdeathsandmarriages/livebirths/datasets/birthsbyparentscharacteristics

Ensembl, https://www.ensembl.org/info/data/ftp/index.html

GenBank, https://www.ncbi.nlm.nih.gov/genbank

gnomAD, https://gnomad.broadinstitute.org/

Human Protein Atlas, www.proteinatlas.org

National Vital Statistics System, https://www.nber.org/research/data/vital-statistics-natality-birth-data.

OMIM, http://www.omim.org/

R Project, https://www.R-project.org/

References

1. Kong, A., Frigge, M.L., Masson, G., Besenbacher, S., Sulem, P., Magnusson, G., Gudjonsson, S.A., Sigurdsson, A., Jonasdottir, A., Jonasdottir, A., et al. (2012). Rate of de novo mutations and the importance of father's age to disease risk. Nature *488*, 471–475. https://doi.org/10.1038/nature11396.
2. Rahbari, R., Wuster, A., Lindsay, S.J., Hardwick, R.J., Alexandrov, L.B., Al Turki, S., Dominiczak, A., Morris, A., Porteous, D., Smith, B., et al. (2016). Timing, rates and spectra of human germline mutation. Nat. Genet. *48*, 126–133. https://doi.org/10.1038/ng.3469.
3. Goldmann, J.M., Wong, W.S., Pinelli, M., Farrah, T., Bodian, D., Stittrich, A.B., Glusman, G., Vissers, L.E., Hoischen, A., Roach, J.C., et al. (2016). Parent-of-origin-specific signatures of de novo mutations. Nat. Genet. *48*, 935–939. https://doi.org/10.1038/ng.3597.
4. Wood, K.A., and Goriely, A. (2022). The impact of paternal age on new mutations and disease in the next generation. Fertil. Steril. *118*, 1001–1012. https://doi.org/10.1016/j.fertnstert.2022.10.017.
5. Sasani, T.A., Pedersen, B.S., Gao, Z., Baird, L., Przeworski, M., Jorde, L.B., and Quinlan, A.R. (2019). Large, three-generation human families reveal post-zygotic mosaicism and variability in germline mutation accumulation. Elife *8*, e46922. https://doi.org/10.7554/eLife.46922.
6. Jonsson, H., Sulem, P., Kehr, B., Kristmundsdottir, S., Zink, F., Hjartarson, E., Hardarson, M.T., Hjorleifsson, K.E., Eggertsson, H.P., Gudjonsson, S.A., et al. (2017). Parental influence on human germline de novo mutations in 1,548 trios from Iceland. Nature *549*, 519–522. https://doi.org/10.1038/nature24018.
7. Goriely, A., and Wilkie, A.O. (2012). Paternal age effect mutations and selfish spermatogonial selection: causes and consequences for human disease. Am. J. Hum. Genet. *90*, 175–200. https://doi.org/10.1016/j.ajhg.2011.12.017.
8. Goriely, A., McVean, G.A., Rojmyr, M., Ingemarsson, B., and Wilkie, A.O. (2003). Evidence for selective advantage of pathogenic FGFR2 mutations in the male germ line. Science *301*, 643–646. https://doi.org/10.1126/science.1085710 301/5633/643.
9. Goriely, A., Hansen, R.M., Taylor, I.B., Olesen, I.A., Jacobsen, G.K., McGowan, S.J., Pfeifer, S.P., McVean, G.A., Rajpert-De Meyts, E., and Wilkie, A.O. (2009). Activating mutations in FGFR3 and HRAS reveal a shared genetic origin for congenital disorders and testicular tumors. Nat. Genet. *41*, 1247–1252. https://doi.org/10.1038/ng.470.
10. Maher, G.J., McGowan, S.J., Giannoulatou, E., Verrill, C., Goriely, A., and Wilkie, A.O. (2016). Visualizing the origins of selfish de novo mutations in individual seminiferous tubules of human testes. Proc. Natl. Acad. Sci. USA *113*, 2454–2459. https://doi.org/10.1073/pnas.1521325113.
11. Maher, G.J., Ralph, H.K., Ding, Z., Koelling, N., Mlcochova, H., Giannoulatou, E., Dhami, P., Paul, D.S., Stricker, S.H., Beck, S., et al. (2018). Selfish mutations dysregulating RAS-MAPK signaling are pervasive in aged human testes. Genome Res. *28*, 1779–1790. https://doi.org/10.1101/gr.239186.118.
12. Giannoulatou, E., McVean, G., Taylor, I.B., McGowan, S.J., Maher, G.J., Iqbal, Z., Pfeifer, S.P., Turner, I., Burkitt Wright, E.M., Shorto, J., et al. (2013). Contributions of intrinsic mutation rate and selfish selection to levels of de novo HRAS mutations in the paternal germline. Proc. Natl. Acad. Sci. USA *110*, 20152–20157. https://doi.org/10.1073/pnas.1311381110.
13. Qin, J., Calabrese, P., Tiemann-Boege, I., Shinde, D.N., Yoon, S.-R., Gelfand, D., Bauer, K., and Arnheim, N. (2007). The molecular anatomy of spontaneous germline mutations in human testes. PLoS Biol. *5*, e224. https://doi.org/10.1371/journal.pbio.0050224.
14. Choi, S.-K., Yoon, S.-R., Calabrese, P., and Arnheim, N. (2008). A germ-line-selective advantage rather than an increased mutation rate can explain some unexpectedly common human disease mutations. Proc. Natl. Acad. Sci. USA *105*, 10143–10148. https://doi.org/10.1073/pnas.0801267105.
15. Choi, S.-K., Yoon, S.-R., Calabrese, P., and Arnheim, N. (2012). Positive selection for new disease mutations in the human germline: evidence from the heritable cancer syndrome multiple endocrine neoplasia type 2B. PLoS Genet. *8*, e1002420. https://doi.org/10.1371/journal.pgen.1002420.
16. Shinde, D.N., Elmer, D.P., Calabrese, P., Boulanger, J., Arnheim, N., and Tiemann-Boege, I. (2013). New evidence for positive selection helps explain the paternal age effect observed in achondroplasia. Hum. Mol. Genet. *22*, 4117–4126. https://doi.org/10.1093/hmg/ddt260.
17. Yoon, S.-R., Choi, S.-K., Eboreime, J., Gelb, B.D., Calabrese, P., and Arnheim, N. (2013). Age-dependent germline mosaicism of the most common noonan syndrome mutation shows the signature of germline selection. Am. J. Hum. Genet. *92*, 917–926. https://doi.org/10.1016/j.ajhg.2013.05.001.
18. Li, M.W.M., Mruk, D.D., and Cheng, C.Y. (2009). Mitogen-activated protein kinases in male reproductive function. Trends Mol. Med. *15*, 159–168. https://doi.org/10.1016/j.molmed.2009.02.002.
19. Wei, B.H., Hao, S.L., and Yang, W.X. (2022). Regulation of spermatogonial stem cell self-renewal and proliferation in mammals. Histol. Histopathol. *37*, 825–838. https://doi.org/10.14670/HH-18-461.
20. Kaplanis, J., Samocha, K.E., Wiel, L., Zhang, Z., Arvai, K.J., Eberhardt, R.Y., Gallone, G., Lelieveld, S.H., Martin, H.C., McRae, J.F., et al. (2020). Evidence for 28 genetic disorders discovered by combining healthcare and research data. Nature *586*, 757–762. https://doi.org/10.1038/s41586-020-2832-5.

21. Le Goff, C., Mahaut, C., Abhyankar, A., Le Goff, W., Serre, V., Afenjar, A., Destrée, A., di Rocco, M., Héron, D., Jacquemont, S., et al. (2011). Mutations at a single codon in Mad homology 2 domain of SMAD4 cause Myhre syndrome. Nat. Genet. *44*, 85–88. https://doi.org/10.1038/ng.1016.
22. Caputo, V., Cianetti, L., Niceta, M., Carta, C., Ciolfi, A., Bocchinfuso, G., Carrani, E., Dentici, M.L., Biamino, E., Belligni, E., et al. (2012). A restricted spectrum of mutations in the SMAD4 tumor-suppressor gene underlies Myhre syndrome. Am. J. Hum. Genet. *90*, 161–169. https://doi.org/10.1016/j.ajhg.2011.12.011.
23. Caputo, V., Bocchinfuso, G., Castori, M., Traversa, A., Pizzuti, A., Stella, L., Grammatico, P., and Tartaglia, M. (2014). Novel SMAD4 mutation causing Myhre syndrome. Am. J. Med. Genet. *164A*, 1835–1840. https://doi.org/10.1002/ajmg.a.36544.
24. Ralph, H.K. (2018). Evaluating Selfish Spermatogonial Selection and its Role in Human Disease (Oxford: DPhil (University of Oxford)).
25. Myhre, S.A., Ruvalcaba, R.H.A., and Graham, C.B. (1981). A new growth deficiency syndrome. Clin. Genet. *20*, 1–5. https://doi.org/10.1111/j.1399-0004.1981.tb01798.x.
26. Lin, A.E., Brunetti-Pierri, N., Lindsay, M.E., Schimmenti, L.A., and Starr, L.J. (1993). In Myhre Syndrome, M.P. Adam, J. Feldman, G.M. Mirzaa, R.A. Pagon, S.E. Wallace, L.J.H. Bean, K.W. Gripp, A. Amemiya, and GeneReviews, eds. https://www.ncbi.nlm.nih.gov/books/NBK425723/.
27. Zhao, M., Mishra, L., and Deng, C.-X. (2018). The role of TGF-β/SMAD4 signaling in cancer. Int. J. Biol. Sci. *14*, 111–123. https://doi.org/10.7150/ijbs.23230.
28. Massagué, J., Seoane, J., and Wotton, D. (2005). Smad transcription factors. Genes Dev. *19*, 2783–2810. https://doi.org/10.1101/gad.1350705.
29. Li, H., Cheng, B., Hu, X., Li, C., Su, J., Zhang, S., Li, L., Li, M., Yang, K., He, S., et al. (2020). The first two Chinese Myhre syndrome patients with the recurrent SMAD4 pathogenic variants: Functional consequences and clinical diversity. Clin. Chim. Acta *500*, 128–134. https://doi.org/10.1016/j.cca.2019.10.006.
30. Alankarage, D., Enriquez, A., Steiner, R.D., Raggio, C., Higgins, M., Milnes, D., Humphreys, D.T., Duncan, E.L., Sparrow, D.B., Giampietro, P.F., et al. (2022). Myhre syndrome is caused by dominant-negative dysregulation of SMAD4 and other co-factors. Differentiation *128*, 1–12. https://doi.org/10.1016/j.diff.2022.09.002.
31. Howe, J.R., Roth, S., Ringold, J.C., Summers, R.W., Järvinen, H.J., Sistonen, P., Tomlinson, I.P., Houlston, R.S., Bevan, S., Mitros, F.A., et al. (1998). Mutations in the SMAD4/DPC4 gene in juvenile polyposis. Science *280*, 1086–1088. https://doi.org/10.1126/science.280.5366.1086.
32. Gallione, C.J., Repetto, G.M., Legius, E., Rustgi, A.K., Schelley, S.L., Tejpar, S., Mitchell, G., Drouin, E., Westermann, C.J.J., and Marchuk, D.A. (2004). A combined syndrome of juvenile polyposis and hereditary haemorrhagic telangiectasia associated with mutations in MADH4 (SMAD4). Lancet *363*, 852–859. https://doi.org/10.1016/S0140-6736(04)15732-2.
33. Blatter, R., Tschupp, B., Aretz, S., Bernstein, I., Colas, C., Evans, D.G., Genuardi, M., Hes, F.J., Hüneburg, R., Järvinen, H., et al. (2020). Disease expression in juvenile polyposis syndrome: a retrospective survey on a cohort of 221 European patients and comparison with a literature-derived cohort of 473 SMAD4/BMPR1A pathogenic variant carriers. Genet. Med. *22*, 1524–1532. https://doi.org/10.1038/s41436-020-0826-1.
34. Wan, R., Feng, J., and Tang, L. (2021). Consequences of Mutations and Abnormal Expression of SMAD4 in Tumors and T Cells. Onco. Targets Theor. *14*, 2531–2540. https://doi.org/10.2147/OTT.S297855.
35. Guo, J., Grow, E.J., Mlcochova, H., Maher, G.J., Lindskog, C., Nie, X., Guo, Y., Takei, Y., Yun, J., Cai, L., et al. (2018). The Adult Human Testis Transcriptional Cell Atlas. Cell Res. *28*, 1141–1157. https://doi.org/10.1038/s41422-018-0099-2.
36. Young, J.C., Wakitani, S., and Loveland, K.L. (2015). TGF-beta superfamily signaling in testis formation and early male germline development. Semin. Cell Dev. Biol. *45*, 94–103. https://doi.org/10.1016/j.semcdb.2015.10.029.
37. Lochab, A.K., and Extavour, C.G. (2017). Bone Morphogenetic Protein (BMP) signaling in animal reproductive system development and function. Dev. Biol. *427*, 258–269. https://doi.org/10.1016/j.ydbio.2017.03.002.
38. Uhlén, M., Fagerberg, L., Hallström, B.M., Lindskog, C., Oksvold, P., Mardinoglu, A., Sivertsson, Å., Kampf, C., Sjöstedt, E., Asplund, A., et al. (2015). Proteomics. Tissue-based map of the human proteome. Science *347*, 1260419. https://doi.org/10.1126/science.1260419.
39. Ran, F.A., Hsu, P.D., Wright, J., Agarwala, V., Scott, D.A., and Zhang, F. (2013). Genome engineering using the CRISPR-Cas9 system. Nat. Protoc. *8*, 2281–2308. https://doi.org/10.1038/nprot.2013.143.
40. Khandwala, Y.S., Zhang, C.A., Lu, Y., and Eisenberg, M.L. (2017). The age of fathers in the USA is rising: an analysis of 168 867 480 births from 1972 to 2015. Hum. Reprod. *32*, 2110–2116. https://doi.org/10.1093/humrep/dex267.
41. Doench, J.G., Fusi, N., Sullender, M., Hegde, M., Vaimberg, E.W., Donovan, K.F., Smith, I., Tothova, Z., Wilen, C., Orchard, R., et al. (2016). Optimized sgRNA design to maximize activity and minimize off-target effects of CRISPR-Cas9. Nat. Biotechnol. *34*, 184–191. https://doi.org/10.1038/nbt.3437.
42. Bernkopf, M., Hunt, D., Koelling, N., Morgan, T., Collins, A.L., Fairhurst, J., Robertson, S.P., Douglas, A.G.L., and Goriely, A. (2017). Quantification of transmission risk in a male patient with a FLNB mosaic mutation causing Larsen syndrome: Implications for genetic counseling in postzygotic mosaicism cases. Hum. Mutat. *38*, 1360–1364. https://doi.org/10.1002/humu.23281.
43. Koelling, N., Bernkopf, M., Calpena, E., Maher, G.J., Miller, K.A., Ralph, H.K., Goriely, A., and Wilkie, A.O.M. (2019). amplimap: a versatile tool to process and analyze targeted NGS data. Bioinformatics *35*, 5349–5350. https://doi.org/10.1093/bioinformatics/btz582.
44. Bernkopf, M., Abdullah, U.B., Bush, S.J., Wood, K.A., Ghaffari, S., Giannoulatou, E., Koelling, N., Maher, G.J., Thibaut, L.M., Williams, J., et al. (2023). Personalized recurrence risk assessment following the birth of a child with a pathogenic de novo mutation. Nat. Commun. *14*, 853. https://doi.org/10.1038/s41467-023-36606-w.
45. Sison, C.P., and Glaz, J. (1995). Simultaneous confidence intervals and sample size determination for multinomial proportions. J. Am. Stat. Assoc. *90*, 1494.
46. Gibson, D.G., Young, L., Chuang, R.-Y., Venter, J.C., Hutchison, C.A., and Smith, H.O. (2009). Enzymatic assembly of DNA molecules up to several hundred kilobases. Nat. Methods *6*, 343–345. https://doi.org/10.1038/nmeth.1318.

47. Motta, M., Fasano, G., Gredy, S., Brinkmann, J., Bonnard, A.A., Simsek-Kiper, P.O., Gulec, E.Y., Essaddam, L., Utine, G.E., Guarnetti Prandi, I., et al. (2021). SPRED2 loss-of-function causes a recessive Noonan syndrome-like phenotype. Am. J. Hum. Genet. *108*, 2112–2129. https://doi.org/10.1016/j.ajhg.2021.09.007.
48. Motta, M., Fidan, M., Bellacchio, E., Pantaleoni, F., Schneider-Heieck, K., Coppola, S., Borck, G., Salviati, L., Zenker, M., Cirstea, I.C., and Tartaglia, M. (2019). Dominant Noonan syndrome-causing LZTR1 mutations specifically affect the Kelch domain substrate-recognition surface and enhance RAS-MAPK signaling. Hum. Mol. Genet. *28*, 1007–1022. https://doi.org/10.1093/hmg/ddy412.
49. Tartaglia, M., Cordeddu, V., Chang, H., Shaw, A., Kalidas, K., Crosby, A., Patton, M.A., Sorcini, M., van der Burgt, I., Jeffery, S., and Gelb, B.D. (2004). Paternal germline origin and sex-ratio distortion in transmission of PTPN11 mutations in Noonan syndrome. Am. J. Hum. Genet. *75*, 492–497. https://doi.org/10.1086/423493.
50. Moloney, D.M., Slaney, S.F., Oldridge, M., Wall, S.A., Sahlin, P., Stenman, G., and Wilkie, A.O. (1996). Exclusive paternal origin of new mutations in Apert syndrome. Nat. Genet. *13*, 48–53. https://doi.org/10.1038/ng0596-48.
51. Glaser, R.L., Jiang, W., Boyadjiev, S.A., Tran, A.K., Zachary, A.A., Van Maldergem, L., Johnson, D., Walsh, S., Oldridge, M., Wall, S.A., et al. (2000). Paternal origin of FGFR2 mutations in sporadic cases of Crouzon syndrome and Pfeiffer syndrome. Am. J. Hum. Genet. *66*, 768–777. https://doi.org/10.1086/302831.S0002-9297(07)64007-5 [pii].
52. Wilkin, D.J., Szabo, J.K., Cameron, R., Henderson, S., Bellus, G.A., Mack, M.L., Kaitila, I., Loughlin, J., Munnich, A., Sykes, B., et al. (1998). Mutations in fibroblast growth-factor receptor 3 in sporadic cases of achondroplasia occur exclusively on the paternally derived chromosome. Am. J. Hum. Genet. *63*, 711–716. https://doi.org/10.1086/302000.S0002-9297(07)61372-X [pii].
53. Rannan-Eliya, S.V., Taylor, I.B., De Heer, I.M., Van Den Ouweland, A.M., Wall, S.A., and Wilkie, A.O. (2004). Paternal origin of FGFR3 mutations in Muenke-type craniosynostosis. Hum. Genet. *115*, 200–207. https://doi.org/10.1007/s00439-004-1151-5.
54. Risch, N., Reich, E.W., Wishnick, M.M., and McCarthy, J.G. (1987). Spontaneous mutation and parental age in humans. Am. J. Hum. Genet. *41*, 218–248.
55. Mayo, J.A., Lu, Y., Stevenson, D.K., Shaw, G.M., and Eisenberg, M.L. (2021). Parental age and preterm birth: a population-based cohort of nearly 3 million California livebirths from 2007 to 2012. J. Perinatol. *41*, 2156–2164. https://doi.org/10.1038/s41372-020-00894-7.
56. Ahn, E.H., and Lee, S.H. (2019). Detection of Low-Frequency Mutations and Identification of Heat-Induced Artifactual Mutations Using Duplex Sequencing. Int. J. Mol. Sci. *20*, 199. https://doi.org/10.3390/ijms20010199.
57. Motta, M., Barresi, S., Pizzi, S., Bifano, D., Lopez Marti, J., Garrido-Pontnou, M., Flex, E., Bruselles, A., Giovannoni, I., Rotundo, G., et al. (2024). RAF1 gene fusions are recurrent driver events in infantile fibrosarcoma-like mesenchymal tumors. J. Pathol. *263*, 166–177. https://doi.org/10.1002/path.6272.
58. Lin, A.E., Michot, C., Cormier-Daire, V., L'Ecuyer, T.J., Matherne, G.P., Barnes, B.H., Humberson, J.B., Edmondson, A.C., Zackai, E., O'Connor, M.J., et al. (2016). Gain-of-function mutations in SMAD4 cause a distinctive repertoire of cardiovascular phenotypes in patients with Myhre syndrome. Am. J. Med. Genet. *170*, 2617–2631. https://doi.org/10.1002/ajmg.a.37739.
59. McRae, J.F., Clayton, S., Fitzgerald, T.W., Kaplanis, J., Prigmore, E., Rajan, D., Sifrim, A., Aitken, S., Akawi, N., Alvi, M., et al. (2017). Prevalence and architecture of de novo mutations in developmental disorders. Nature *542*, 433–438. https://doi.org/10.1038/nature21062.
60. Lin, A.E., Scimone, E.R., Thom, R.P., Balaguru, D., Kinane, T.B., Moschovis, P.P., Cohen, M.S., Tan, W., Hague, C.D., Dannheim, K., et al. (2024). Emergence of the natural history of Myhre syndrome: 47 patients evaluated in the Massachusetts General Hospital Myhre Syndrome Clinic (2016-2023). Am. J. Med. Genet. e63638. https://doi.org/10.1002/ajmg.a.63638.
61. Slezak, R., Smigiel, R., Obersztyn, E., Pollak, A., Dawidziuk, M., Wiszniewski, W., Bekiesinska-Figatowska, M., Rydzanicz, M., Ploski, R., and Gawlinski, P. (2021). Further Delineation of Phenotype and Genotype of Primary Microcephaly Syndrome with Cortical Malformations Associated with Mutations in the WDR62 Gene. Genes 12, 594. https://doi.org/10.3390/genes12040594.
62. Grantham, R. (1974). Amino acid difference formula to help explain protein evolution. Science *185*, 862–864. https://doi.org/10.1126/science.185.4154.862.
63. Costantini, A., Guasto, A., and Cormier-Daire, V. (2023). TGF-beta and BMP Signaling Pathways in Skeletal Dysplasia with Short and Tall Stature. Annu. Rev. Genom. Hum. Genet. *24*, 225–253. https://doi.org/10.1146/annurev-genom-120922-094107.
64. Attisano, L., and Wrana, J.L. (2002). Signal Transduction by the TGF-β Superfamily. Science *296*, 1646–1647. https://doi.org/10.1126/science.1071809.
65. Archambeault, D.R., and Yao, H.H. (2014). Loss of smad4 in Sertoli and Leydig cells leads to testicular dysgenesis and hemorrhagic tumor formation in mice. Biol. Reprod. *90*, 62. https://doi.org/10.1095/biolreprod.113.111393.
66. Hao, X.X., Chen, S.R., Tang, J.X., Li, J., Cheng, J.M., Jin, C., Wang, X.X., and Liu, Y.X. (2016). Selective deletion of Smad4 in postnatal germ cells does not affect spermatogenesis or fertility in mice. Mol. Reprod. Dev. *83*, 615–623. https://doi.org/10.1002/mrd.22664.
67. Bush, S.J., Nikola, R., Han, S., Suzuki, S., Yoshida, S., Simons, B.D., and Goriely, A. (2024). Adult Human, but Not Rodent, Spermatogonial Stem Cells Retain States with a Foetal-like Signature. Cells *13*, 742. https://doi.org/10.3390/cells13090742.
68. Itman, C., Mendis, S., Barakat, B., and Loveland, K.L. (2006). All in the family: TGF-beta family action in testis development. Reproduction *132*, 233–246. https://doi.org/10.1530/rep.1.01075.
69. Zuo, Q., Jin, K., Zhang, Y., Song, J., and Li, B. (2017). Dynamic expression and regulatory mechanism of TGF-beta signaling in chicken embryonic stem cells differentiating into spermatogonial stem cells. Biosci. Rep. *37*, BSR20170179. https://doi.org/10.1042/BSR20170179.
70. Loveland, K.L., and Hime, G. (2005). TGFbeta superfamily members in spermatogenesis: setting the stage for fertility in mouse and Drosophila. Cell Tissue Res. *322*, 141–146. https://doi.org/10.1007/s00441-005-0008-0.
71. Puglisi, R., Montanari, M., Chiarella, P., Stefanini, M., and Boitani, C. (2004). Regulatory role of BMP2 and BMP7 in

spermatogonia and Sertoli cell proliferation in the immature mouse. Eur. J. Endocrinol. *151*, 511–520. https://doi.org/10.1530/eje.0.1510511.

72. Zhao, G.Q., Chen, Y.X., Liu, X.M., Xu, Z., and Qi, X. (2001). Mutation in Bmp7 exacerbates the phenotype of Bmp8a mutants in spermatogenesis and epididymis. Dev. Biol. *240*, 212–222. https://doi.org/10.1006/dbio.2001.0448.
73. Pellegrini, M., Grimaldi, P., Rossi, P., Geremia, R., and Dolci, S. (2003). Developmental expression of BMP4/ALK3/SMAD5 signaling pathway in the mouse testis: a potential role of BMP4 in spermatogonia differentiation. J. Cell Sci. *116*, 3363–3372. https://doi.org/10.1242/jcs.00650.
74. Tan, K., Song, H.W., Thompson, M., Munyoki, S., Sukhwani, M., Hsieh, T.C., Orwig, K.E., and Wilkinson, M.F. (2020). Transcriptome profiling reveals signaling conditions dictating human spermatogonia fate in vitro. Proc. Natl. Acad. Sci. USA *117*, 17832–17841. https://doi.org/10.1073/pnas.2000362117.
75. Moreno, S.G., Attali, M., Allemand, I., Messiaen, S., Fouchet, P., Coffigny, H., Romeo, P.H., and Habert, R. (2010). TGFbeta signaling in male germ cells regulates gonocyte quiescence and fertility in mice. Dev. Biol. *342*, 74–84. https://doi.org/10.1016/j.ydbio.2010.03.007.
76. Moustakas, A., and Heldin, C.H. (2005). Non-Smad TGF-beta signals. J. Cell Sci. *118*, 3573–3584. https://doi.org/10.1242/jcs.02554.
77. Yue, J., and Mulder, K.M. (2000). Activation of the mitogen-activated protein kinase pathway by transforming growth factor-beta. Methods Mol. Biol. *142*, 125–131. https://doi.org/10.1385/1-59259-053-5:125.
78. Roelen, B.A., Cohen, O.S., Raychowdhury, M.K., Chadee, D.N., Zhang, Y., Kyriakis, J.M., Alessandrini, A.A., and Lin, H.Y. (2003). Phosphorylation of threonine 276 in Smad4 is involved in transforming growth factor-beta-induced nuclear accumulation. Am. J. Physiol. Cell Physiol. *285*, C823–C830. https://doi.org/10.1152/ajpcell.00053.2003.
79. Park, D.S., Yoon, G.H., Kim, E.Y., Lee, T., Kim, K., Lee, P.C., Chang, E.J., and Choi, S.C. (2020). Wip1 regulates Smad4 phosphorylation and inhibits TGF-beta signaling. EMBO Rep. *21*, e48693. https://doi.org/10.15252/embr.201948693.
80. Demagny, H., Araki, T., and De Robertis, E.M. (2014). The tumor suppressor Smad4/DPC4 is regulated by phosphorylations that integrate FGF, Wnt, and TGF-beta signaling. Cell Rep. *9*, 688–700. https://doi.org/10.1016/j.celrep.2014.09.020.
81. Watanabe, H., de Caestecker, M.P., and Yamada, Y. (2001). Transcriptional cross-talk between Smad, ERK1/2, and p38 mitogen-activated protein kinase pathways regulates transforming growth factor-beta-induced aggrecan gene expression in chondrogenic ATDC5 cells. J. Biol. Chem. *276*, 14466–14473. https://doi.org/10.1074/jbc.M005724200.
82. Mulder, K.M. (2000). Role of Ras and Mapks in TGFbeta signaling. Cytokine Growth Factor Rev. *11*, 23–35. https://doi.org/10.1016/s1359-6101(99)00026-x.

The American Journal of Human Genetics, Volume *111*

Supplemental information

SMAD4 mutations causing Myhre syndrome are under positive selection in the male germline

Katherine A. Wood, R Spencer Tong, Marialetizia Motta, Viviana Cordeddu, Eleanor R. Scimone, Stephen J. Bush, Dale W. Maxwell, Eleni Giannoulatou, Viviana Caputo, Alice Traversa, Cecilia Mancini, Giovanni B. Ferrero, Francesco Benedicenti, Paola Grammatico, Daniela Melis, Katharina Steindl, Nicola Brunetti-Pierri, Eva Trevisson, Andrew OM. Wilkie, Angela E. Lin, Valerie Cormier-Daire, Stephen RF. Twigg, Marco Tartaglia, and Anne Goriely

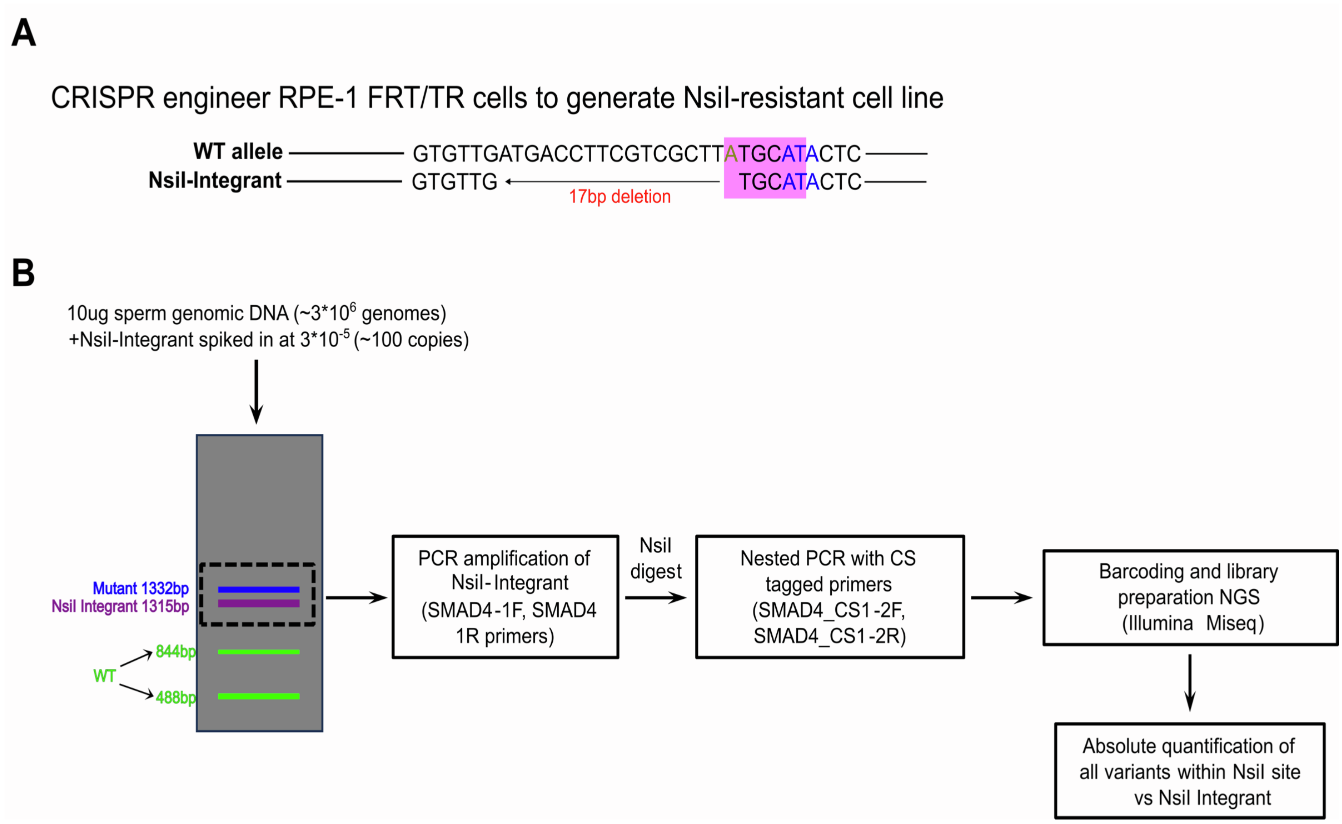

Figure S1. Overview of the NsiI RED-PCR assay to enrich mutant sequences at position c.1494_1499 of *SMAD4.* (**A**) Sequence context of the NsiI-integrant following CRISPR-Cas engineering, a heterozygous clone with a 17-bp deletion (c.1478_1494del). The NsiI site is boxed in purple and the Ile500 codon is highlighted in blue. The engineered mutant allele disrupts the NsiI restriction site (the final base of the deletion encompasses the first base of the NsiI cut site (position c.1494A) making the CRISPR allele resistant to enzyme digestion. (**B**) Overview of the RED-PCR design involving a double NsiI and PstI digestion of 10 µg of human gDNA (blood or sperm), supplemented with ~100 copies of mutant allele of the NsiI-Integrant clone, separation of the NsiI-resistant fragments by gel electrophoresis, followed by two rounds of PCR amplification, barcoding of individual samples and ultra-deep Illumina sequencing.

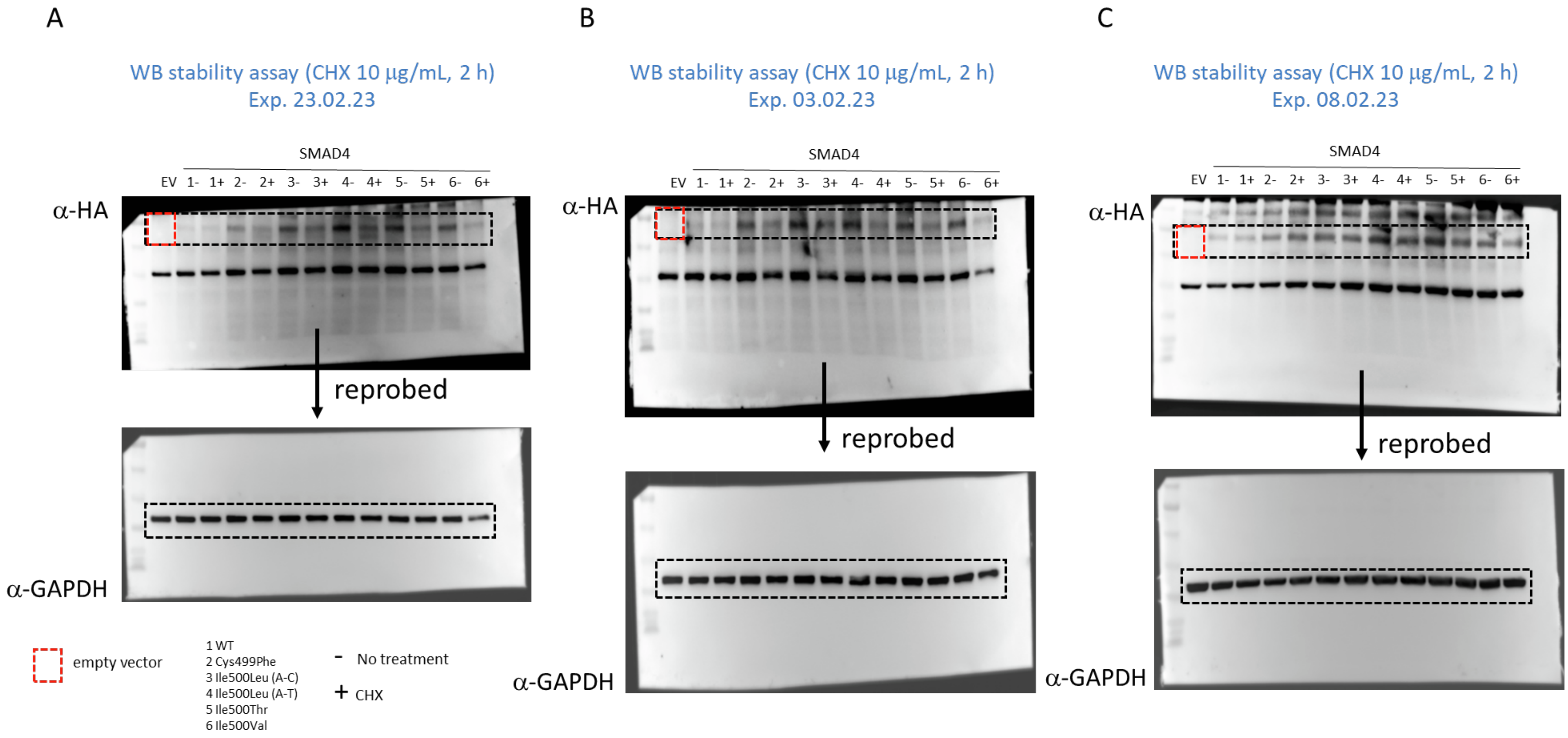

Figure S2. Uncropped pictures of the western blotting membranes used for the densitometric analysis of SMAD4 protein levels (SMAD4 stability assay) presented in **Figure 3B**. (**A**), (**B**) and (**C**) refer to the three independent experiments performed. For each membrane, the extent of the cropped region is shown. For experimental conditions and antibody specifications please refer to the Materials and Methods section.

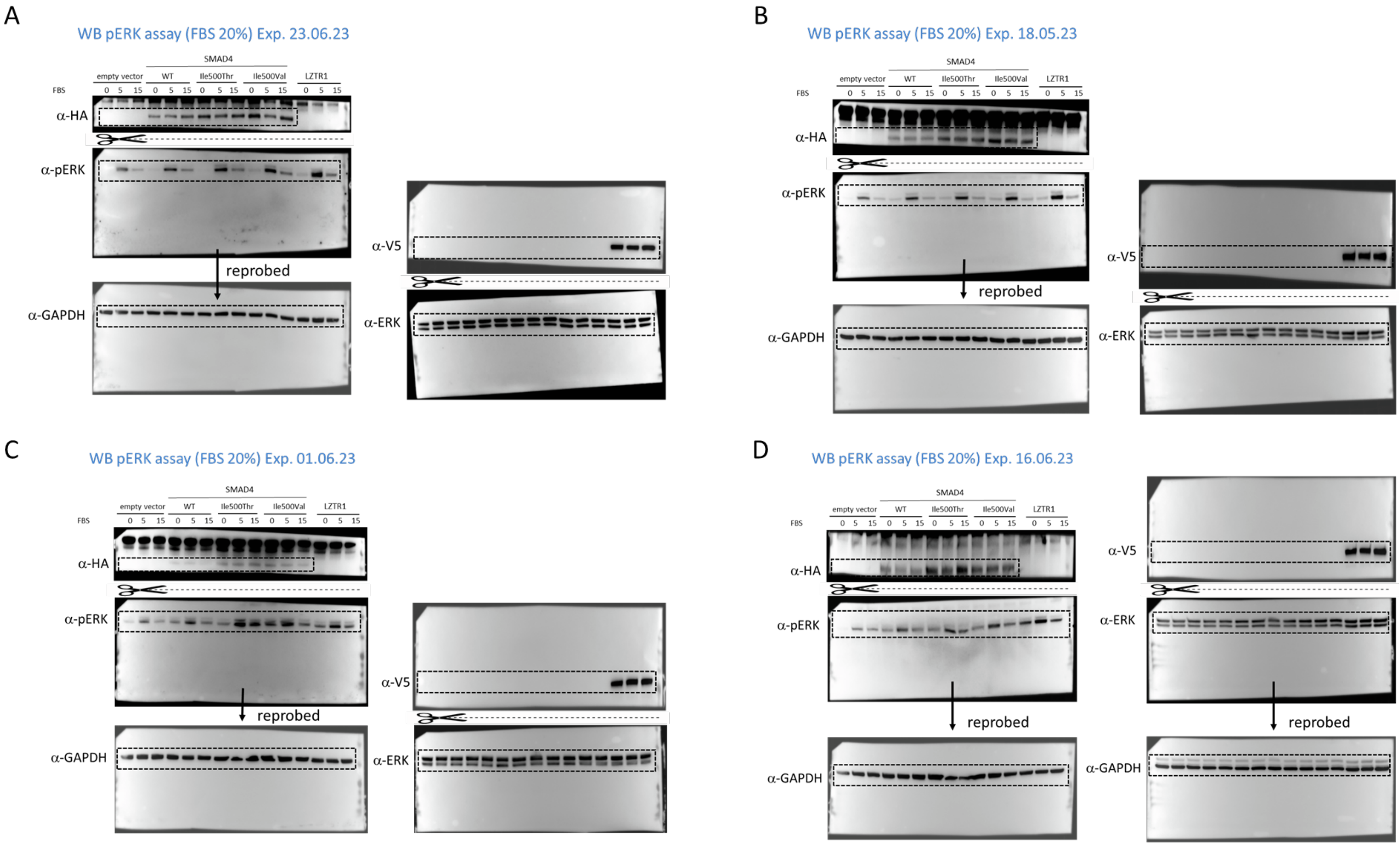

Figure S3. Uncropped pictures of western blotting membranes used for densitometric analysis of ERK1/2 phosphorylation levels at Thr202/Thr185 and Tyr204/Tyr187 (pERK assay) presented in **Figure 3C**. (**A**), (**B**), (**C**) and (**D**) refer to the four independent experiments performed. For each membrane, the extent of the cropped region is shown. Membranes were cut to enable blotting for multiple antibodies. For experimental conditions and antibodies specifications please refer to the Materials and Methods section.

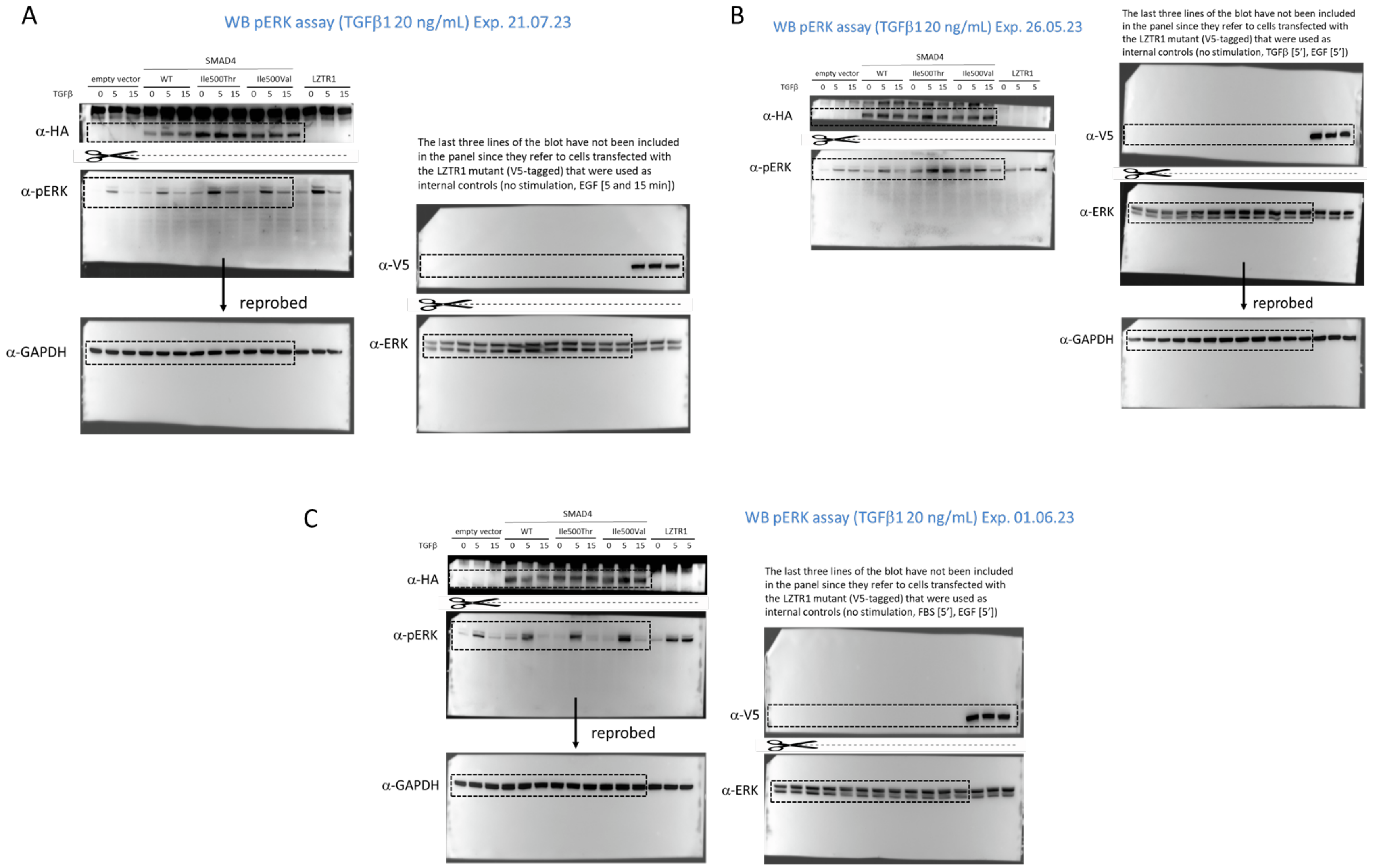

Figure S4. Uncropped pictures of western blotting membranes used for densitometric analysis of ERK1/2 phosphorylation levels at Thr202/Thr185 and Tyr204/Tyr187 (pERK assay) (TGFβ1 stimulation) presented in **Figure 3D**. (**A**), (**B**) and (**C**) refer to the three independent experiments performed. For each membrane, the extent of the cropped region is shown. Membranes were cut to enable blotting for multiple antibodies. For experimental conditions and antibodies specifications please refer to the Materials and Methods section.

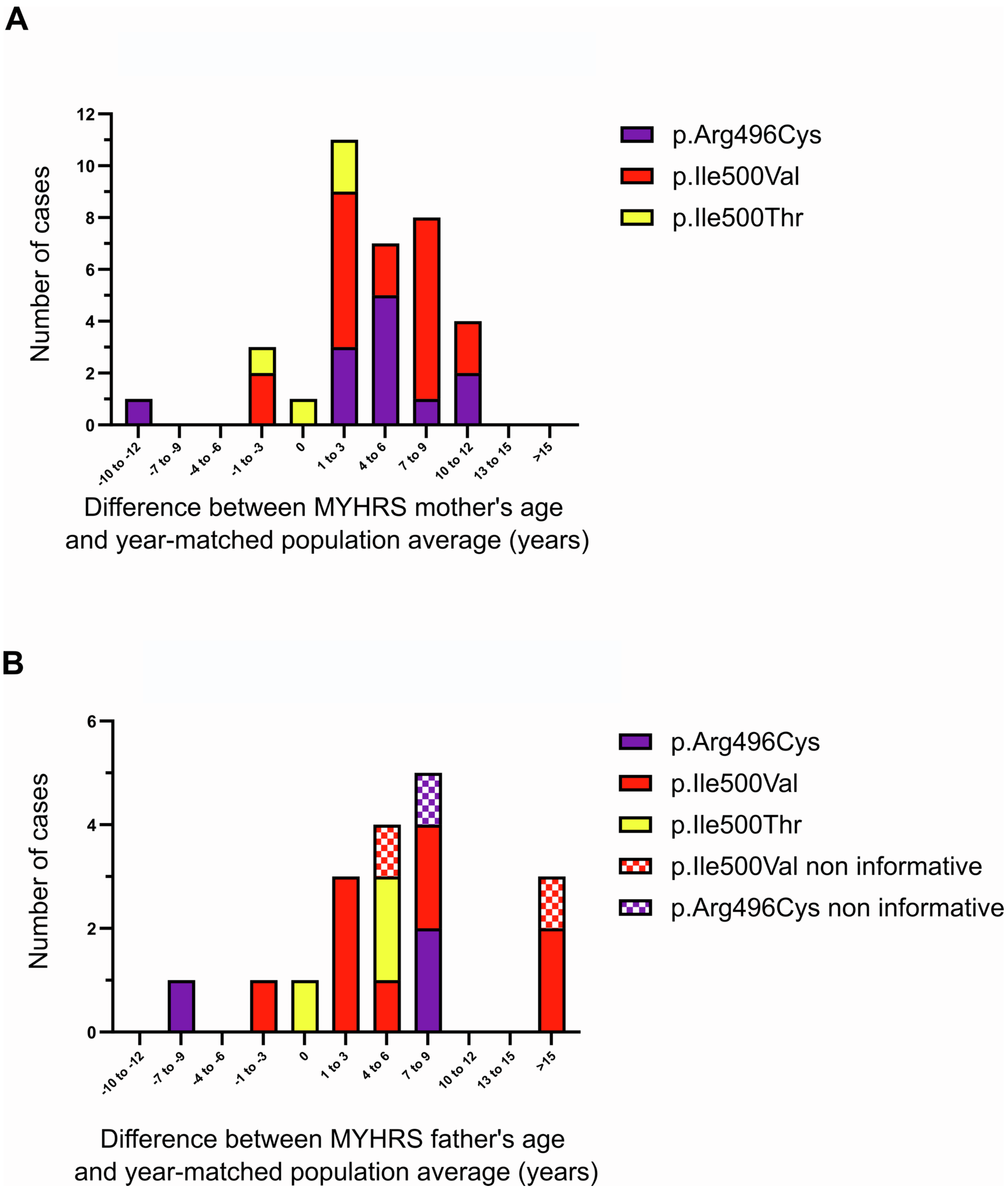

Figure S5. Parental age effects in MYHRS cohorts. (**A**) Maternal age excess observed for probands with confirmed MYHRS *de novo* variants (as indicated on the figure) for a USA cohort (n = 35). (**B**) Excess paternal age observed in MYHRS families for a European (EU) cohort with confirmed parental origin of the *de novo* mutation. The X-axis represents the difference of paternal ages between MYHRS fathers and the year-match fatherhood average in England and Wales (UK). Note we had access to age data for 17 EU MYHRS families and among them 14 had proven paternal origin excess age (plain colors). The 3 uninformative families are indicated in dotted colors (see **Table S2**).

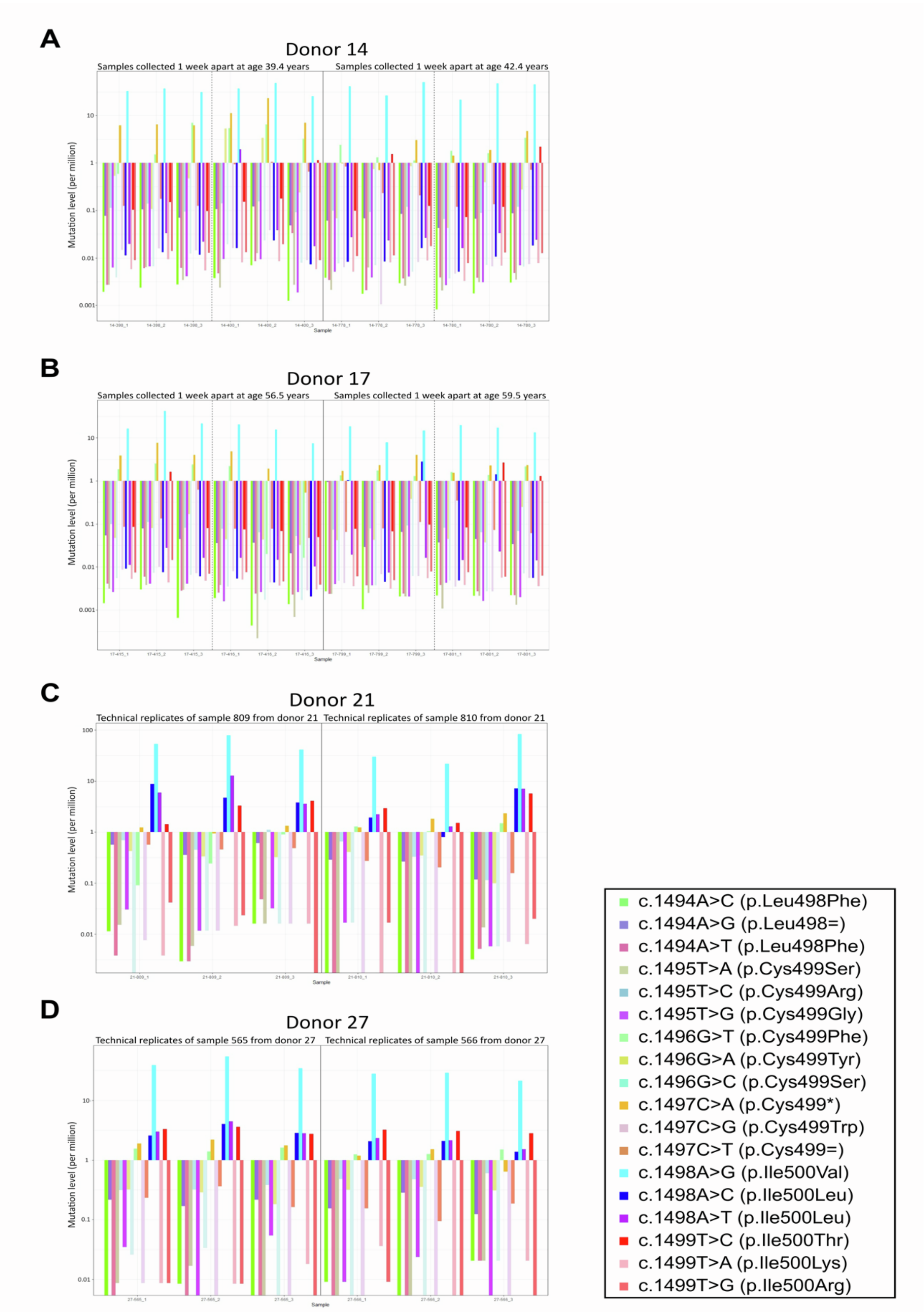

Figure S6. Reproducibility of the NsiI RED_PCR assay. The mutation levels of each substitution at *SMAD4* c.1494_1499 were quantified by the NsiI RED_PCR assay for independent biological semen samples from 4 different donors. For each sample, 3 technical replicates (underscores 1-3 on the X-axis) were processed in parallel. (**A-B**) For donors 14 and 17, four sets of samples are shown, two samples were taken a week apart and another set of two samples from the same donor three years later. (**C-D**) For donors 21 and 27, two sets of samples were collected within a week of each other (figure relates to data presented in **Table S4**).

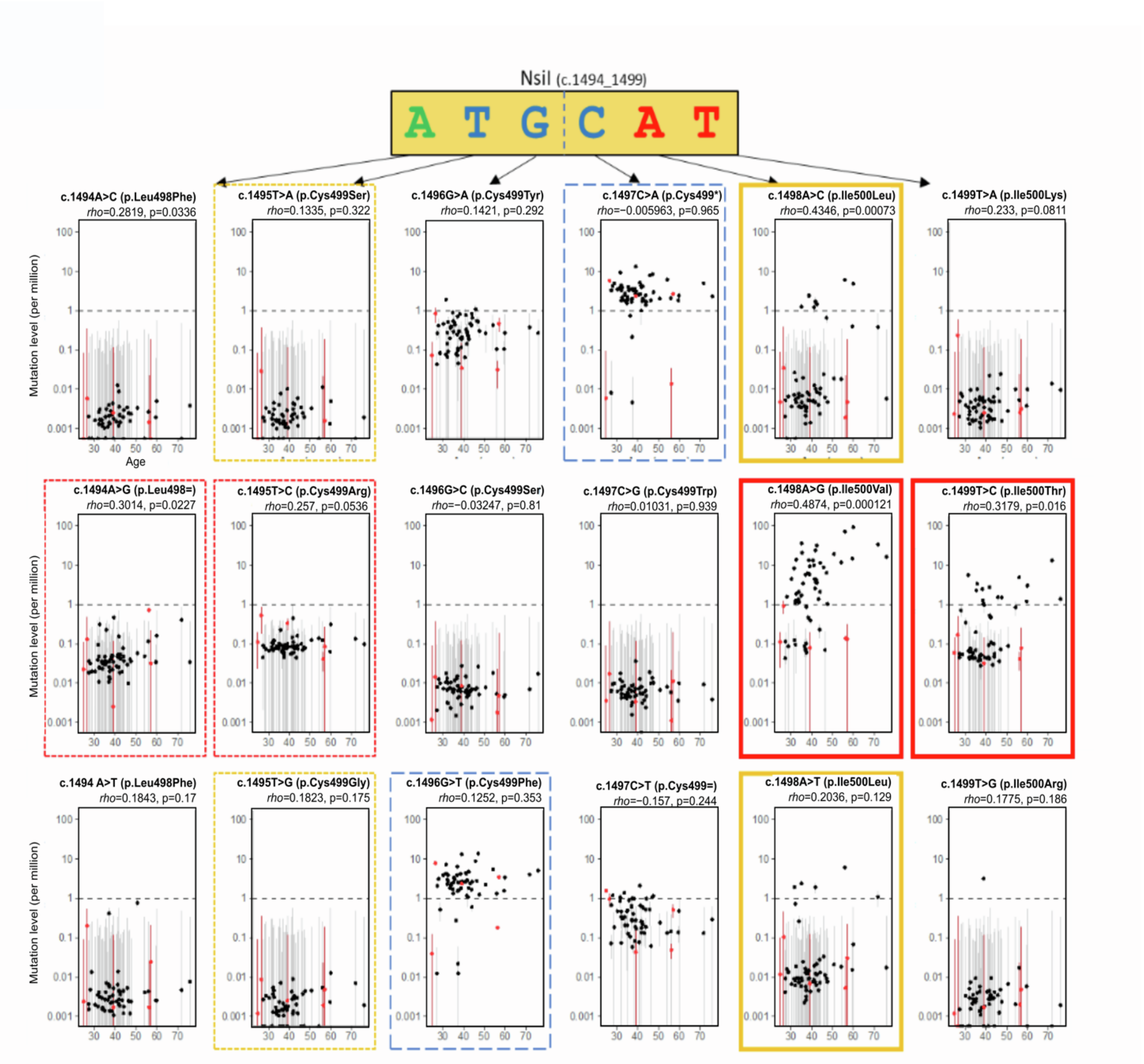

Figure S7. Quantification of mutation levels within the NsiI site in sperm and blood samples. Each of the 18 substitutions at *SMAD4* c.1494_1499 from 57 sperm (black) and 5 blood (red) samples (from donors aged 24.8 - 75.9 years, age shown on X-axis) are shown. Panels have been organized according to the substitutions within the NsiI site (orange box at the top, note the palindromic nature of the site, indicated by the dotted blue line within the box). Estimated mutation levels following the RED_PCR enrichment strategy are plotted as the mean of 3 independent technical replicates and their 95% binomial CI on a log10 scale (Y-axis). The dotted horizontal black lines represent the theoretical 10-6 limit of detection of the RED_PCR assay. The mutations found to be enriched in sperm have been boxed (red box for the two known MYHRS variants c.1488A>G and c.1499T>C) and orange for the two substitutions encoding p.Ile500Leu). Moreover, their cognate/reciprocal positions within the palindromic site are highlighted by dotted boxes of matching colors. Also note the p.Cys499Phe/Stop substitutions (dotted blue boxes) are cognate (c.1496G>T | c.1497C>A) substitutions and show a symmetrical pattern. A rho value representing the correlation between mutation level and donor age is shown above each panel. Individual data are provided in **Table S5** and summarized in **Table S6**.
